# A continuation method for spatially discretized models with nonlocal interactions conserving size and shape of cells and lattices

**DOI:** 10.1007/s00285-020-01534-6

**Published:** 2020-09-21

**Authors:** Shin-Ichiro Ei, Hiroshi Ishii, Makoto Sato, Yoshitaro Tanaka, Miaoxing Wang, Tetsuo Yasugi

**Affiliations:** 1 Department of Mathematics, Faculty of Science, Hokkaido University, Sapporo, 060-0810 Japan; 2 Mathematical Neuroscience Unit, Institute for Frontier Science Initiative, Kanazawa University, 13-1 Takaramachi, Kanazawa-shi, Ishikawa 920-8640 Japan; 3 Department of Complex and Intelligent Systems, Future University Hakodate, 116-2 Kamedanakano-cho, Hakodate, Hokkaido 041-8655 Japan

**Keywords:** Continuation method, Nonlocal interactions, Spatially discretized model, Singular limit analysis, Delta–Notch signaling, 35B36, 35A35, 92B05, 35Q92

## Abstract

In this paper, we introduce a continuation method for the spatially discretized models, while conserving the size and shape of the cells and lattices. This proposed method is realized using the shift operators and nonlocal operators of convolution types. Through this method and using the shift operator, the nonlinear spatially discretized model on the uniform and nonuniform lattices can be systematically converted into a spatially continuous model; this renders both models point-wisely equivalent. Moreover, by the convolution with suitable kernels, we mollify the shift operator and approximate the spatially discretized models using the nonlocal evolution equations, rendering suitable for the application in both experimental and mathematical analyses. We also demonstrate that this approximation is supported by the singular limit analysis, and that the information of the lattice and cells is expressed in the shift and nonlocal operators. The continuous models designed using our method can successfully replicate the patterns corresponding to those of the original spatially discretized models obtained from the numerical simulations. Furthermore, from the observations of the isotropy of the Delta–Notch signaling system in a developing real fly brain, we propose a radially symmetric kernel for averaging the cell shape using our continuation method. We also apply our method for cell division and proliferation to spatially discretized models of the differentiation wave and describe the discrete models on the sphere surface. Finally, we demonstrate an application of our method in the linear stability analysis of the planar cell polarity model.

## Introduction

**Fig. 1:**
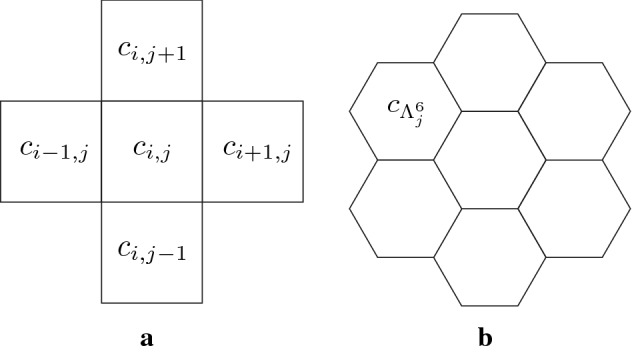
Schematic figures of the square **(a)** and hexagonal **(b)** lattices with indexes

The development of multicellular organisms is regulated by intercellular communication and signaling pathways of various types. These include diffusible proteins acting as ligands and cell membrane proteins communicating with the neighboring cells. In the last fifty years, approaches comprising mathematical models and numerical simulations have been extensively used to understand the mechanisms underlying the biological phenomena. It is a common practice to divide a region of interest either into square or hexagonal elements representing cells, as shown in Fig. 1; this allows for the discrete spatial independent variables to be used. We also assume that the unknown dependent variables of the model are uniform on the lattices. With these preconditions, we model the phenomena on the lattices mathematically. In this paper, we label the mathematical models with the discrete spatial independent variable as discrete models, and the ones with the continuous spatial independent variable as the continuous models. Modeling the phenomena on the divided lattices often demonstrates good reproducibility and presents good agreement with experimental results.

**Fig. 2:**
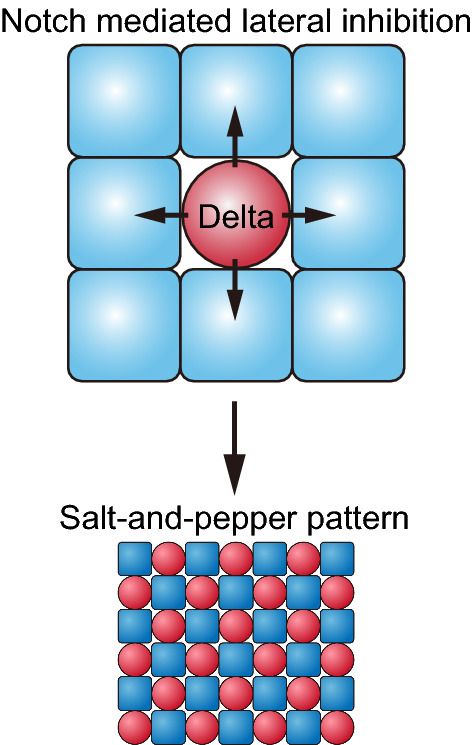
Schematic figures of interaction of Delta–Notch signaling and a salt-and-pepper pattern

One of the examples in which the cellular interaction is conserved in various organisms is the Delta–Notch signaling. The intercellular communication in this signaling is based on the informational exchange between neighboring cells (Collier et al. 1996; Sato et al. 2013; Yasugi et al. 2008). The function of the Delta–Notch signaling is known as lateral inhibition. During neural development of the fly embryo, binding of the Delta ligand to the Notch receptor suppresses the expression of achaete-scute complex (AS-C) proneural genes. On the contrary, AS-C genes induce Delta expression. Consequently, signal-sending cells demonstrate a high level of the AS-C genes, while signal-receiving cells express low level of the AS-C genes. During embryogenesis, neuroepithelial cells (NEs) that express high Delta and AS-C differentiate into neural progenitor cells. In contrast, the surrounding cells express low levels of AS-C genes and differentiate into non-neuronal cells. In accordance with these interactions between the neighboring cells, the expression patterns of Delta and Notch activation show a salt-and-pepper like pattern that distinguish the neuronal cells from the non-neuronal ones, as shown in Fig. 2. Information regarding discreteness, such as the size and shape of each cell, affects the entire pattern in the developmental process, therefore, modeling in the framework of the discrete model is compatible with the phenomenon described (Collier et al. 1996; Lehotzky and Zupanc 2019; Sato et al. 2016). A discrete model shows good reproducibility of the experimental results for the differentiation propagation in the developing fly brain (Sato et al. 2016; Tanaka et al. 2018). Using the described type of discrete model for the Delta–Notch interaction, the salt-and-pepper pattern appearance and regulated differentiation propagation in the fly brain have been explained (Collier et al. 1996; Jörg et al. 2019; Sato et al. 2016; Tanaka et al. 2018). It is well known that the function of Delta–Notch signaling is diverse and Notch activation shows several different patterns. For example, Notch activation oscillates in the segmentation in vertebrates and progresses unidirectionally in the fly optic lobe development Kageyama et al. (2012).

Another good example of a biological system that utilizes discrete modeling is the planar cell polarity (PCP) (Adler 2002; Goodrich and Strutt 2011). The intercellular proteins and membrane in the cells of the fly wing interact with each other, among the neighboring cells, and they are localized asymmetrically. Owing to this asymmetric localization, the direction of the epithelial hair in the wing of a fly is determined Ayukawa et al. (2014). It is reported that the discrete model proposed by this paper can reproduce the biological experiments of the PCP.

The analytical study of the discrete models was further conducted to specify the function of the intercellular interactions and the discreteness in the dynamics. The analytical results for the discrete type of reaction diffusion systems have been reported in Bates et al. (2001), Chow (2003). The results related to the traveling wave solutions in the system of the discrete models are reported in Guo et al. (2019), Hupkes and Sandstede (2010), Straatman and Hupkes (2019).

Although the discrete models are useful in describing the abovementioned behavior and dynamics phenomenologically, the analysis of discrete models is rather difficult in general, and the technique of analyzing a discrete model is poor compared to the analysis of the continuous models. For example, as the discrete models usually comprise numerous unknown variables, it is usually difficult to compute the eigenvalues in case of higher-dimensional space. Thus, the methods of analysis for the partial differential equation are being reconstructed, such that they are applicable to the discrete models (Chow 2003). Moreover, discrete models are not compatible with the description of regional expansion caused by cell division and the three-dimensional information. In order to overcome the difficulties mentioned, the limit of the cell and lattice size is often set to zero, and the differential operator is derived. There are some examples of the continuation. The discrete models to prion dynamics and coagulation-fragmentation processes were investigated mathematically through the continuation method using the piece-wise constant functions (Crampin et al. 1999; Laurençot and Mischler 2002). In these papers, the continuous models were derived on the lattices with sufficiently small length. By taking the limit of the lattice size, the convergence is rigorously shown, and the integro-differential equations were derived.

However, the method taking the limit of the lattice size to zero may cause a problem because the patterns caused by the spatially discretized structures, such as lattice and cell membrane, sometimes disappear in the continuous models. In this paper, we questioned if it is possible to convert a discrete model into a continuous model while retaining the size and shape of cells and lattices. In the light of this question, we propose a novel continuation method for the spatially discretized models, while conserving the size and shape of cells and lattices. In our proposed method, we perform the continuation part of the process by introducing the shift operators, instead of deriving the differential operators. Thus, the nonlinear discrete models can be systematically converted into continuous models with spatially discretized structures. Moreover, by reducing the shift operators using the integral operators of the convolution type with suitable kernels, we propose a nonlocal evolution equation that can approximate the solution of the spatially discrete model, for the application to the biological experiments and mathematical analysis. The approximation of nonlinear discrete models by nonlocal evolution equations in the one-dimensional periodic region is assured by singular limit analysis. In the continuous models, the information of the size and shape of cells and lattice was reflected in the part of shift operator and the kernel in the convolutions. Furthermore, we confirmed the isotropy of the Delta–Notch signaling system for the irregularly shaped cell in the fly brain. According to the biological results, we propose a radially symmetric kernel in the nonlocal evolution equation for discrete models and prove the effectiveness of the kernel by replicating the spatially discretized patterns in the numerical simulations. As a result of the description using nonlocal evolution equation, we model the cell division and proliferation in the discrete model for the wave of the differentiation and extend the model to the sphere surface. Moreover, we show that linear stability analysis can be performed by the continuation method.

This paper is organized as follows: In Sect. 2, we first introduce the concept of our continuation method by modifying the general discrete model. Our continuation method is characterized by the combination of the shift operator and the Friedrichs mollifiers as the convolution kernel based on the lattice shape. In Sect. 2.2 we state the result of the singular limit analysis of the discrete models and nonlocal evolution equations in the general form of the spatial interactions. In Sect. 2.3 we explain that our method can be extended to the discrete models on nonuniform lattice. In Sect. 2.5.3, we introduce the radially symmetric kernel for the averaged shape of the cells, based on the results of the real biological experiments for Delta–Notch signaling in the fly brain. In Sect. 3, we show the results of numerical simulations in biological applications of the continuation method: the proneural wave in the fly brain and planar cell polarity in the fly wing. Our results indicate that the continuation method using shift operators and integral operators can be applicable for diverse multicellular systems.

## Continuation method with shift and convolution operator for discrete models

### Scalar equation in one-dimensional space

In this section, we explain the concept of the continuation method, while retaining the shape and size of cells and lattices.

First, we describe the continuation method applied on a typical discrete model containing the intercellular interaction terms and the reaction term. In this paper, we do not distinguish between the spatial and intercellular interactions. Suppose *N* cells of the uniform length $l>0$ are packed in the one-dimensional space, then the following discrete model is considered:

1 $$\begin{aligned} u_{i,t} = f( u_{i-1}, \ u_{i}, \ u_{i+1} )+g( u_{i}), \quad t>0 , \quad i=1, \ldots , N, \end{aligned}$$

where $u_{i} = u_{i}(t)$ denotes the concentration or density of some substances on the $i\hbox {th}$ cell $c_i$ at time $t>0$, $f: {\mathbb {R}}^3 \rightarrow {\mathbb {R}}$ is the function corresponding to the intercellular interactions, and $g: {\mathbb {R}}\rightarrow {\mathbb {R}}$ is the function for the reactions.

Setting the one-dimensional space as

$$\begin{aligned} {\mathbb {T}}:=[0, Nl], \end{aligned}$$

we impose the periodic boundary condition $$u_0(t)=u_{N}(t)$$, and $$ u_1(t)=u_{N+1}(t)$$. The linear intercellular interaction can be defined as:

2 $$\begin{aligned} f(u_{-1}, u_0, u_1) := \sum _{i=-1}^1 a_iu_i =a_{-1}u_{-1}+ a_0 u_0 + a_1u_1, \end{aligned}$$

where $a_i, \ (i=-1,0,1)$ are constants. The typical examples of the function *f* are diffusion and lateral inhibition such as the Delta–Notch interaction given by

3 $$\begin{aligned} f_{\varDelta }( u_{i-1}, u_{i}, u_{i+1} )&= \frac{u_{i-1} -2 u_{i} + u_{i+1} }{l^2}, \end{aligned}$$

4 $$\begin{aligned} f_{\text {lat}}( u_{i-1}, u_{i+1} )&= \frac{ - u_{i-1} - u_{i+1} }{ {2 } }, \end{aligned}$$

where the denominator of $f_{\text {lat}}$ is the total number of neighboring cells referred from Collier et al. (1996), and the sign of the lateral inhibition $f_{\text {lat}}$ can be changed in the system. If the dynamics of $u_i$ is more influenced by the other cells than by the neighboring cells, *f* becomes the nonlocal interactions.

As introduced in Doumic et al. (2009), Laurençot and Mischler (2002), we will utilize the piece-wise constant functions for our continuation method. For equation (1) with $i=1, \ldots , N$ on each cell $c_i$, we define the characteristic function as

5 $$\begin{aligned} \chi _{c_i}(x)= \left\{ \begin{array}{cl} 1 &{}\quad \text {if} \ x \in c_i,\\ 0 &{}\quad \text {otherwise}, \end{array} \right. \end{aligned}$$

and also we define

$$\begin{aligned} u(x,t) : = \sum _{i=1 }^{N } u_{i}(t) \chi _{c_i}(x) \end{aligned}$$

at position $x \in {\mathbb {T}}$ and at time $t>0$. For the continuous method of the discrete model, we set the following assumption:

A1 $$\begin{aligned} \begin{aligned}&\text { For any }N\text { there exists a unique global solution } u(x,t) \in C([0, T], L^1({\mathbb {T}})) \\&\text { of }(1). \\ \end{aligned} \end{aligned}$$

As in Proposition 2 and “Appendix B”, the existence and uniqueness of the global solution of (1) is shown for specified functions *f* and *g*. Changing the variable in the *i*th equation (1) by multiplying the unknown function $u_i$ by the characteristic function $\chi _{c_i}(x)$, and adding $u_i(t)\chi _{c_i}(x)$ with respect to $i=1, \cdots , N$, we have

$$\begin{aligned} u_{t} = f \left( \sum _{i=1 }^{N } u_{i-1} (t) \chi _{c_i} (x), \ u, \ \sum _{i=1 }^{N } u_{i+1} (t) \chi _{c_i} (x) \right) +g( u ). \end{aligned}$$

As we can compute

6 $$\begin{aligned} \sum _{i=1 }^{N } u_{i+j} \chi _{c_i}(x) = \sum _{i=1 }^{N } u_{i} \chi _{c_i}(x + jl ) =u( x+ jl, t ) \end{aligned}$$

for $j=0, \pm 1, \cdots , \pm N$, we obtain

7 $$\begin{aligned} u_{t} = f( u ( x- l, t ), \ u, \ u(x + l, t ) )+g( u ). \end{aligned}$$

As the spatially independent variable is continuous, the discrete model (1) is successfully converted into a continuous model. The equation of (7) is point-wisely equivalent to the equation (1). Thus, if the initial conditions of equation (1) and (7) are the same, the solutions are equivalent as described by the following remark:

#### Remark 1

Using the initial data of discrete model $\{ u_i(0)\}_{i=1}^N$, and imposing the initial data as $u_0(x):=u(x,0)= \sum _{i=1 }^{N } u_{i}(0) \chi _{c_i}(x)$, the solution of continuous model (7) is equivalent to that of the discrete model (1).

Furthermore, to apply the continuous model to the experiments and analyze conveniently, we approximate the shift operator using the convolution on the mollifier. We define the shift operator as follows:

$$\begin{aligned} \tau _{l} u(x):= u(x+l). \end{aligned}$$

The shift operator is regarded as the convolution of the shifted Dirac Delta function $\delta _l : = \tau _l \delta =\delta (x+l)$, and we can describe the model (7) as follows:

$$\begin{aligned} u_{t} = f( u * \delta _{-l} , \ u, \ u *\delta _l )+g( u ). \end{aligned}$$

Here we suppose that the Dirac Delta function is periodic with *Nl*, i.e., $$\delta (x) = \delta (x + Nl)$$, and we define the convolution $$k *v $$ with respect to *x* in $${\mathbb {T}}$$ as

$$\begin{aligned} (k*v)(x) := \int _{{\mathbb {T}}} k(x-y) v(y) dy, \end{aligned}$$

where ${\mathbb {T}}$ can be replaced with a given region in this paper. Setting the Friedrichs mollifier with a small parameter $0<\varepsilon \ll 1$ as

$$\begin{aligned} \rho _{\varepsilon }(x) :=\frac{1}{\varepsilon }\rho \Big (\frac{1}{\varepsilon }x \Big ), \quad \rho (x) := \left\{ \begin{alignedat}{2} C_0 \exp \left( -\frac{1}{1- \left| x \right| ^2} \right) ,&\quad \left| x \right| < 1,\\ 0,&\quad \left| x \right| \ge 1 \end{alignedat} \right. \end{aligned}$$

with a constant for the normalization of integration $C_0>0$, we assume that $\rho _\varepsilon$ is also periodic with *Nl*. We use the symbol ${\varvec{\rho }}_\varepsilon$ for the mollifier in higher-dimensional case. Approximating the Dirac Delta function by the mollifier $\rho _{\varepsilon }(x)$, we have

8 $$\begin{aligned} u_{t}^{\varepsilon } = f( u^{\varepsilon }*\rho _{\varepsilon , -l}, \ u^{\varepsilon }, \ u^{\varepsilon }*\rho _{\varepsilon , l} )+g( u^{\varepsilon } ), \end{aligned}$$

where the shifted mollifier is given by $\rho _{\varepsilon , l}:=\rho _\varepsilon (x+l)$, and we denote the unknown variable by $u^{\varepsilon }(x,t)$ as the solution of this equation depends on $\varepsilon$. If the intercellular interaction *f* is linear, we derive the typical nonlocal evolution equation by summarizing the kernel of the convolution as follows:

$$\begin{aligned}&f( u^{\varepsilon }*\rho _{\varepsilon , -l}, \ u^{\varepsilon }, \ u^{\varepsilon }*\rho _{\varepsilon , l} )+g( u^{\varepsilon } )\\&=a_{-1}u^{\varepsilon }*\rho _{\varepsilon , -l} + a_0 u^{\varepsilon } + a_1 u^{\varepsilon }*\rho _{\varepsilon , l} +g( u^{\varepsilon } )\\&= (a_{-1} \rho _{\varepsilon , -l}+ a_1 \rho _{\varepsilon , l} ) * u^{\varepsilon }+ a_0 u^{\varepsilon } + g( u^{\varepsilon } )\\&= K*u^{\varepsilon }+ a_0 u^{\varepsilon } + g( u^{\varepsilon } ), \end{aligned}$$

where we put the kernel as $K=a_{-1} \rho _{\varepsilon , -l} + a_1 \rho _{\varepsilon , l}$. Consequently, we have the following nonlocal evolution equation:

9 $$\begin{aligned} u_{t}^{\varepsilon } = K*u^{\varepsilon }+ a_0 u^{\varepsilon } + g( u^{\varepsilon } ). \end{aligned}$$

**Fig. 3:**
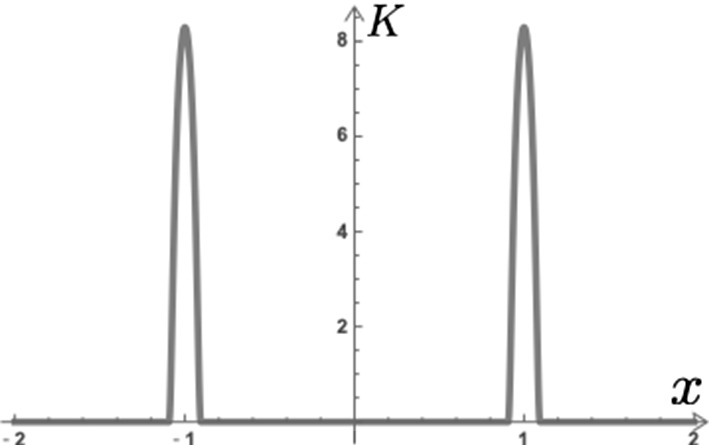
Profile of the kernel *K* of $f_{\varDelta }$. The parameters are $a_{-1}=a_1=1$, $l=1$, and $\varepsilon =0.1$

Such type of a nonlocal evolution has been analyzed in numerous papers (Bates et al. 1997; Coville and Dupaigne 2007). Figure 3 shows the profile of the kernel *K* for $f_{\varDelta }$.

**Fig. 4:**
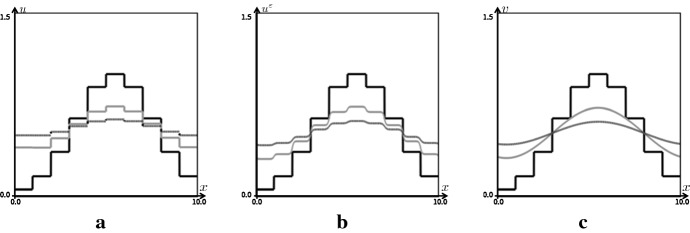
Results of numerical simulations of heat equations in form of model (7), model (8) and the original heat equation. Periodic boundary condition is imposed in ${\mathbb {T}}=[0,10]$, and the parameters are $l=1.0$, $dx=1/200$, $f=f_{\varDelta }$, and $\varepsilon =0.1$ in the mollifier, respectively. The black, gray, and dotted curves correspond to the profile of solution at $t=0$, $t=2.0$ and $t=4.0$. **a** $u_{t} = f_{\varDelta }( u ( x- l, t ), \ u, \ u(x + l, t ) )$, **b** $u_t^\varepsilon =f_{\varDelta }(u^\varepsilon *\rho _{\varepsilon , -l}, \ u^\varepsilon , \ u^\varepsilon *\rho _{\varepsilon , l} )$, **c** $v_t = v_{xx}$

Figure 4 shows the results of the numerical simulations for both continuous and discrete heat equations fed with the spatially discretized initial data.

As in Fig. 4a, it is observed that the solution of the equation with shift operator is not continuous until the solution attains the steady-state in the numerical simulation. On the contrary, as in Fig. 4b, it is observed that the solution of the equation with the mollifier becomes continuous before the solution attains the steady-state in the numerics.

#### Remark 2

If *f* and *g* are linear, and if $u^\varepsilon =u* \rho _\varepsilon$, $u^\varepsilon$ becomes the solution of (7). This is owing to the linearity of the convolution operator, which can be described as follows; using the convolution of the mollifier in the equation (7), we have

$$\begin{aligned} u_{t}* \rho _\varepsilon = f( u ( x- l, t )* \rho _\varepsilon , \ u* \rho _\varepsilon , \ u(x + l, t )* \rho _\varepsilon )+g( u* \rho _\varepsilon ). \end{aligned}$$

We compute as follows

$$\begin{aligned} u_{t}^\varepsilon = f( u^\varepsilon ( x- l, t ) , \ u^\varepsilon , \ u^\varepsilon (x + l, t ) )+g( u^\varepsilon ) \end{aligned}$$

thereby satisfying Eq. (7).

Furthermore, our proposed method is consistent with the continuation method where the cell limit was set to 0 or lattice size was set to *l*, as we can derive the differential operator by setting the limit $l \rightarrow +0$ after applying our continuation method.

#### Remark 3

If *f* is equal to $f_{\varDelta }$, we see that

$$\begin{aligned} \begin{aligned} f_{\varDelta }&=\frac{u(x-l,t) -2 u + u(x+l, t)}{ l^2 } \rightarrow u_{xx} \end{aligned} \end{aligned}$$

as $$l \rightarrow +0$$.

Even if the intercellular interaction is nonlocal, which means it is affected by not only the neighboring cells but also the other cells, our continuation method is applicable to the discrete model, in a similar way. A discrete model in which intercellular interactions are influenced by the cells other than neighboring cells, is given as follows: 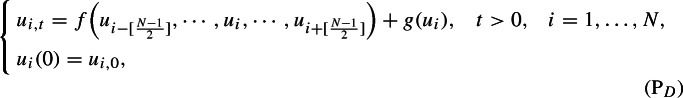 where $[\cdot ]$ is the Gauss’s symbol, and $f : {\mathbb {R}}^{ 2[N-1/2] + 1} \rightarrow {\mathbb {R}}$ is a function corresponding to the interaction here. If *f* is linear, the function *f* is generally defined as

10 $$\begin{aligned} f(u_{ - [\frac{N-1}{2} ] }, \cdots , u_0, \cdots , u_{ [\frac{N-1}{2} ] } )= \sum _{ i = - [\frac{N-1}{2} ] }^{ [\frac{N-1}{2} ] } a_i u_i, \end{aligned}$$

where $\{ a_i \}_{i=-[(N-1)/2]} ^ { [(N-1)/2] }$ are constants. Following the calculation in (6), we derive the equivalent continuous model as follows: 
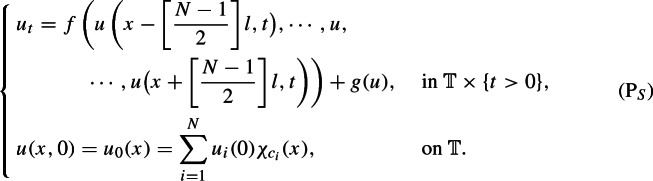
 By describing the nonlocal interactions using the convolution of the mollifier $\rho _\varepsilon$, the kernel is provided by

11 $$\begin{aligned} K=\sum _{j=- [\frac{N-1}{2} ] , j\ne 0}^{ [\frac{N-1}{2} ] } a_j \tau _{jl}\rho _\varepsilon , \end{aligned}$$

and thus, the nonlocal evolution, which can approximate the solution of ($\hbox {P}_S$), is given as follows: 
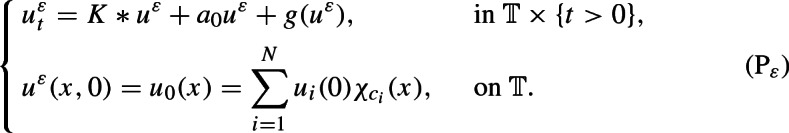


### Singular limit analysis

In this subsection, we will explain that the solution of ($\hbox {P}_\varepsilon$) is sufficiently close to that of ($\hbox {P}_S$) in $L^2({\mathbb {T}})$ by singular limit analysis. As the interaction *f* with the form in ($\hbox {P}_D$) includes the case of intercellular interaction in the discrete model, we deal with the equations ($\hbox {P}_S$) and ($\hbox {P}_\varepsilon$) in this analysis. We firstly assume that *f* is the form of (10). For the condition *g*, we assume that there exist positive constants $g_0, g_2, g_4$ and a nonnegative constant $g_1, g_3$ such that for $u,v \in {\mathbb {R}}$,

A2 $$\begin{aligned}&g(u)u \le -g_0 |u|^{p+1} + g_1|u|^3 + g_2|u|^2, \end{aligned}$$

A3 $$\begin{aligned}&| g_u(u) +pg_0| u |^{p-1} | \le g_3|u| + g_4, \end{aligned}$$

A4 $$\begin{aligned}&p\ge 3 \text { or } g_1=g_3=0 \text { if } 2<p<3. \end{aligned}$$

A typical example of *g* is $g(u)=u(1-u^2)$, where $g_0=g_2=g_4=1$, $g_1=g_3=0$ and $p=3$.

First, we calculate the fundamental solution of the ($\hbox {P}_S$) without the reaction term *g*(*u*).

#### Proposition 1

The fundamental solution of $u_t = \displaystyle { \sum _{ j = - [\frac{N-1}{2} ] }^{ [\frac{N-1}{2} ] } a_j u(x {+} jl , t) }$ with initial datum $u_0(x)= \sum _{i=1 }^{N } u_{i}(0) \chi _{c_i}(x)$ is given by

$$\begin{aligned} u(x, t ) = \sum _{j=1}^N \sum _{k=1}^N p_k e^{ \lambda _k t} \omega ^{kj} \chi _{c_j}(x), \end{aligned}$$

12 $$\begin{aligned} \lambda _k = \sum _{ h = -[\frac{N-1}{2}] }^{[\frac{N-1}{2}]} a_h \omega ^{ h k }, \end{aligned}$$

where $k=1, \ldots , N$, $\omega = e^{\frac{ 2 \pi }{N} i}$, *i* is imaginary number and $\{p_k\}_{k=1}^{ N }$ are constants determined by the initial data.

Furthermore, if $a_j = a_{-j}, \ (j=1, \ldots , [ \frac{N-1}{2} ] )$ the fundamental solution can be written by

$$\begin{aligned} u(x, t )&= \sum _{j=1}^N \sum _{k=1}^N q_k e^{ \lambda _k t} \Big ( \cos \Big ( \frac{ 2 k j \pi }{ N}\Big ) + \sin \Big ( \frac{ 2 k j \pi }{ N}\Big ) \Big ) \chi _{c_j}(x),\\ \lambda _k&= a_0 + 2 \sum _{ h = 1 }^{[\frac{N-1}{2} ]} a_h \cos \Big ( \frac{ 2\pi {h} k}{N} \Big ), \end{aligned}$$

where $\{q_k\}_{k=1}^{ N }$ are real constants determined by the initial data.

The proof is in “Appendix B”. Since the equation ($\hbox {P}_S$) is equivalent to ($\hbox {P}_D$), and the associated matrix from *f* of the system ($\hbox {P}_D$) is the cyclic, the eigenvalue and the eigenvector can be calculated.

Next, we have the uniqueness and global existence of the solutions of ($\hbox {P}_S$) and ($\hbox {P}_\varepsilon$).

#### Proposition 2

Assume that *f* is given by (10), and $\sup _{j \in \{ -\left[ ( N-1)/ 2 \right] , \ldots , \left[ ( N-1)/2 \right] \} } | a_j | < \infty$. There exists a unique solution $u \in C[ (0, \infty ), L^\infty ({\mathbb {T}}) ]$ of ($\hbox {P}_S$) with an initial datum $u_0 \in L^{\infty }({\mathbb {T}})$. Moreover,

13 $$\begin{aligned} \sup _{ 0\le t<\infty } \Vert u(\cdot ,t) \Vert _{L^\infty ({\mathbb {T}})} < C_1 \end{aligned}$$

where $C_1$ is a positive constant.

#### Proposition 3

Assume that *K* is given by (11), and $\sup _{j \in \{ -\left[ ( N-1)/ 2 \right] , \ldots , \left[ ( N-1)/2 \right] \} } | a_j | < \infty$. There exists a unique solution $u^\varepsilon \in C[ (0, \infty ), L^\infty ({\mathbb {T}}) ]$ of ($\hbox {P}_\varepsilon$) with an initial datum $u_0 \in L^\infty ({\mathbb {T}})$. Moreover,

14 $$\begin{aligned} \sup _{ 0\le t<\infty } \Vert u^\varepsilon (\cdot ,t) \Vert _{L^\infty ({\mathbb {T}})} < C_2 \end{aligned}$$

where $C_2$ is a positive constant independent of $\varepsilon$.

The proves are in “Appendix B”. We note that each global solution of ($\hbox {P}_S$) and ($\hbox {P}_\varepsilon$) is in $L^2({\mathbb {T}})$ due to $L^\infty ({\mathbb {T}}) \subset L^2({\mathbb {T}})$. Thus, we have global boundedness in $L^2({\mathbb {T}})$ as

$$\begin{aligned} \sup _{0\le t<\infty }\left\| u(\cdot , t) \right\| _{L^2({\mathbb {T}})}< C_3, \quad \sup _{0\le t<\infty }\left\| u^\varepsilon (\cdot , t) \right\| _{L^2({\mathbb {T}})} < C_4 \end{aligned}$$

from the estimations (13) and (14).

For the solution of the model ($\hbox {P}_S$) and ($\hbox {P}_\varepsilon$), we have the following error estimate. Setting the error between the solution of ($\hbox {P}_S$) and ($\hbox {P}_\varepsilon$) as

$$\begin{aligned} U^{\varepsilon }(x, t) := u^{\varepsilon }(x,t) - u(x, t), \end{aligned}$$

we have the following convergence result.

#### Theorem 1

Suppose the same assumptions of Propositions 2 and 3. Let *u*(*x*, *t*) and $u^\varepsilon (x,t)$ be solutions of ($\hbox {P}_S$) and ($\hbox {P}_\varepsilon$) with initial datum $u(x,0)=u^\varepsilon (x,0) =u_0(x)=\sum _{i=1 }^{N } u_{i}(0) \chi _{c_i}(x) \in L^{\infty }({\mathbb {T}})$, respectively. Then

$$\begin{aligned} \sup _{0<t<T}\Vert U^{\varepsilon } (\cdot ,t)\Vert _{L^2({\mathbb {T}})} \le \sqrt{ \frac{ C_6 }{C_5}\Big ( e^{ C_5 T} -1 \Big ) } \sup _{|y| < \varepsilon , t>0 } \Vert \tau _y u - u \Vert _{L^2({\mathbb {T}})}, \end{aligned}$$

where $C_5$ and $C_6$ are positive constants independent of $\varepsilon$. Thus, we have

$$\begin{aligned} \Vert U^{\varepsilon } (\cdot , t) \Vert _{ L^2({\mathbb {T}}) } \rightarrow 0 \end{aligned}$$

as $\varepsilon \rightarrow +0$ for any $0< t < T$.

The calculation of the energy estimate is put in “Appendix B”. From this estimation, the solution of the continuous model (8) converges to that of (7) in $L^2({\mathbb {T}})$ space as $\varepsilon$ tends to 0. This implies the solution of nonlocal evolution equation can approximate the solution of discrete model.

#### Corollary 1

Assume that *f* is global Lipschitz continuous, i.e., there exists a positive constant *L* such that

15 $$\begin{aligned} \begin{aligned}&\left| f \left( u_{ - [\frac{N-1}{2} ] }, \cdots , u_0, \cdots , u_{ [\frac{N-1}{2} ] } \right) - f \left( v_{ - [\frac{N-1}{2} ] }, \cdots , v_0, \cdots , v_{ [\frac{N-1}{2} ] } \right) \right| \\&\qquad \le L \sum _{j=-[\frac{N-1}{2} ]}^{[\frac{N-1}{2} ]} |u_j -v_j|, \end{aligned} \end{aligned}$$

and $f( \varvec{0} )=0, \ (\varvec{0} \in {\mathbb {R}}^N)$. Then Proposition 2, Proposition 3, and Theorem 1 hold.

Here, ($\hbox {P}_\varepsilon$) has the term $f(u^\varepsilon *\tau _{ -[ N-1 /2] l} \rho _\varepsilon ,\cdots , u^\varepsilon ,\cdots , u^\varepsilon *\tau _{ [ N-1 /2] l} \rho _\varepsilon )$ instead of $K*u^\varepsilon -a_0u^\varepsilon$. The typical example of above *f* is in the model of the PCP (38). By using the inequality (15), the proof is followed by that of Proposition 2, Proposition 3, and Theorem 1.

### Nonuniform lattice in one-dimensional space

In this subsection, we introduce that our continuation method is applicable to the discrete models on nonuniform lattices by adding some conditions as a remark. Labeling the $$i\hbox {th}$$ cell as $$c_i, \ (i=1,\ldots , N)$$ with the nonuniform length $$l_i>0$$, we suppose *N* cells are packed in the one-dimensional space. Let $$u_{i} = u_{i}(t)$$ be the concentration or density of some substances on $$c_i$$ at time $$t>0$$. Imposing the periodic boundary condition $$u_0(t)=u_{N}(t)$$, and $$ u_1(t)=u_{N+1}(t)$$ with $$l_0 = l_N$$, and $$l_1 = l_{N+1}$$, we consider the following discrete model in this subsection:

16 $$\begin{aligned} u_{i,t} = f( u_{i-1}, \ u_{i}, \ u_{i+1} )+g( u_{i}), \quad t>0, \quad i=1, \ldots , N, \end{aligned}$$

where the definitions of *f* and *g* are same as those in (1), and the initial data are given by $\{ u_i(0)\}_{i=1}^N$. For the length $l_i$, we define the following functions:

$$\begin{aligned} l_i (x)&:= l_{i-1} + \frac{ l_i - l_{i-1} }{ l_{i} }\Big ( x - \sum _{j=1}^{i-1} l_j \Big ), \\ r_i (x)&:= l_i + \frac{ l_{i+1} - l_i }{ l_{i} }\Big ( x - \sum _{j=1}^{i-1} l_j \Big ) \end{aligned}$$

for $$x \in c_i$$. These functions map *x* to the division point in left and right neighboring lattices comparing the position *x* in a lattice and left and right neighboring lattices, respectively. We note that if $$l_i = l$$ for all $$i = 1, \ldots , N$$, we have $$ l_i (x) = r_i (x) = l$$ for any $$x \in c_i$$. Using the characteristic function (5), we define the piecewise constant function for the shift as follows

$$\begin{aligned} l (x) := \sum _{i=1}^N l_i (x)\chi _{c_i}(x), \quad r (x) := \sum _{i=1}^N r_i (x)\chi _{c_i}(x) \end{aligned}$$

for $x \in \Big [ 0, \sum _{i=1}^N l_i \Big ]$ in this subsection. Similarly to Sect. 2.1, we set

$$\begin{aligned} u(x,t) : = \sum _{i=1 }^{N } u_{i}(t) \chi _{c_i}(x). \end{aligned}$$

Changing the variable in the $i\hbox {th}$ equation (16) by multiplying the unknown function $u_i$ by the characteristic function $\chi _{c_i}(x)$, and adding $u_i(t)\chi _{c_i}(x)$ with respect to $i=1, \cdots , N$, we have

$$\begin{aligned} u_{t} = f \left( \sum _{i=1 }^{N } u_{i-1} (t) \chi _{c_i} (x), \ u, \ \sum _{i=1 }^{N } u_{i+1} (t) \chi _{c_i} (x) \right) +g( u ). \end{aligned}$$

As we can compute that

$$\begin{aligned}&\sum _{i=1 }^{N } u_{i-1} (t) \chi _{c_i}(x) = \sum _{i=1 }^{N } u_{i} (t) \chi _{c_i}(x - l (x) ) =u( x - l (x), t ), \\&\sum _{i=1 }^{N } u_{i+1} (t) \chi _{c_i}(x) = \sum _{i=1 }^{N } u_{i} (t) \chi _{c_i}(x + r(x) ) =u( x+ r(x), t ), \end{aligned}$$

we obtain

17 $$\begin{aligned} u_{t} = f( u ( x - l (x), t ), \ u, \ u(x + r(x), t ) )+g( u ), \quad \text {in} \ \Big [ 0, \sum _{i=1}^N l_i \Big ] \times \{t>0\}. \end{aligned}$$

If the initial datum is given by $u_0(x):=u(x,0)= \sum _{i=1 }^{N } u_{i}(0) \chi _{c_i}(x)$, the solution of continuous model (16) is equivalent to that of the discrete model (17).

For the mathematical analysis, the function *l*(*x*) and *r*(*x*) can be rendered continuous by using mollifier. We see that *l*(*x*) and *r*(*x*) belong to $L^1\Big ( \Big [0, \sum _{i=1}^N l_i \Big ] \Big )$. Setting

$$\begin{aligned} l^\varepsilon (x) := (l * \rho _\varepsilon ) (x), \quad r^\varepsilon (x) := ( r * \rho _\varepsilon ) (x), \end{aligned}$$

we propose an approximation model to (17) as

18 $$\begin{aligned} u^\varepsilon _{t} = f( u^\varepsilon ( x - l^\varepsilon (x), t ), \ u^\varepsilon , \ u^\varepsilon (x + r^\varepsilon (x), t ) )+g( u^\varepsilon ), \quad \text {in} \ \Big [ 0, \sum _{i=1}^N l_i \Big ] \times \{t>0\}. \end{aligned}$$

Similarly to Sect. 2.1, we approximate the nonuniform shift operators by the convolutions. We assume (A1) for the function $u^\varepsilon$ in (18). We can rewrite (18) in the convolution form as

$$\begin{aligned} u^\varepsilon _{t} = f( u^\varepsilon * \delta _{ - l^\varepsilon (x) } , \ u^\varepsilon , \ u^\varepsilon * \delta _{ r^\varepsilon (x) } )+g( u^\varepsilon ). \end{aligned}$$

Recalling the function $\rho _{\varepsilon , l} = \rho _{\varepsilon } (x+l)$ in (8), we have

$$\begin{aligned} u^{\varepsilon ,\eta }_{t} = f( u^{\varepsilon ,\eta } * \rho _{\eta , - l^\varepsilon (x) } , \ u^{\varepsilon ,\eta }, \ u^{\varepsilon ,\eta } * \rho _{ \eta , r^\varepsilon (x) } )+g( u^{\varepsilon ,\eta } ), \end{aligned}$$

where $0 < \eta \ll 1$ is a constant, and $u^{\varepsilon ,\eta } * \rho _{ \eta , -l^\varepsilon (x) } = \int _{0}^{ \sum _{i=1}^N l_i } u^{\varepsilon ,\eta } (y,t) \rho _{\eta } (x - y - l^\varepsilon (x) ) dy$. If *f* is the linear function defined in (2), we obtain the nonlocal evolution equation

19 $$\begin{aligned} u^{\varepsilon ,\eta }_{t}&= K (x, \cdot )* u^{\varepsilon ,\eta } + a_0 u^{\varepsilon ,\eta } + g( u^{\varepsilon ,\eta } ), \end{aligned}$$

where the kernel is given by

$$\begin{aligned} K(x,y) : = a_{-1} \rho _{\eta } ( y - l^\varepsilon (x) ) + a_1 \rho _{\eta } ( y + r^\varepsilon (x) ). \end{aligned}$$

With $\varepsilon >0$ and $\eta >0$ the kernel *K*(*x*, *y*) is differentiable. As introduced above, the discrete models on nonuniform lattice can be rendered continuous models. The nonlocal evolution equation (19) is expected to approximate the solutions of that of the original discrete models on nonuniform lattices. The error estimations, the analysis and the application to this proposed model is one of our future works.

### System in one-dimensional space

In the case of system we can perform the continuation method similarly to Sect. 2.1. We will explain the method by using the typical reaction diffusion system and Delta–Notch signaling system which are often used for the mathematical modeling in the successive sub-subsections.

#### Reaction diffusion system

First, we explain the typical reaction diffusion system in the one-dimensional space with periodic boundary conditions. Let $u_i=u_i(t)$ and $v_i=v_i(t)$ be the concentration of the diffusive substances on the uniform cells or lattices $c_i, \ (i=1, \ldots , N)$, respectively. The reaction diffusion system in the framework of the discrete model can be described as follows:

20 $$\begin{aligned} \left\{ \begin{aligned} u_{i,t}&= d_u f_{ \varDelta }( u_{i-1}, \ u_i, \ u_{i+1} ) + g_1(u_i, v_i),\\ v_{i,t}&= d_v f_{ \varDelta }( v_{i-1}, \ v_i, \ v_{i+1} )+ g_2(u_i, v_i), \end{aligned} \right. \quad t>0, \quad i=1, \ldots , N, \end{aligned}$$

where $d_u, d_v>0$ are the diffusion coefficients, $f_{ \varDelta }$ is defined by (3), and $g_1, g_2:{\mathbb {R}}^2 \rightarrow {\mathbb {R}}$ are the functions for reactions in this sub-subsection. For this equation, setting the variables at position $x \in {\mathbb {T}}$ and at time $t>0$ as

$$\begin{aligned} u(x,t) : = \sum _{i=1 }^{N } u_{i}(t) \chi _{c_i}(x), \quad v(x,t) : = \sum _{i=1 }^{N } v_{i}(t) \chi _{c_i}(x), \end{aligned}$$

we derive the reaction diffusion system performed our continuation method as follows:

21 $$\begin{aligned} \left\{ \begin{aligned} u_{t}&= d_u f_{ \varDelta }( u( x-l, t ), \ u(x,t), \ u( x+l, t ) ) + g_1( u, v ),\\ v_{t}&= d_v f_{ \varDelta }( v( x-l, t ), \ v(x,t), \ v( x+l, t ) ) + g_2( u, v ). \end{aligned} \right. \quad \text {in } {\mathbb {T}}\times \{t>0\}. \end{aligned}$$

Similarly to the previous subsection, this above equation is point-wisely equivalent to the equation (20). Indeed, if the initial conditions of (20) and (21) are same, the solutions of (20) and (21) are equivalent.

Approximating the shift operator by the mollifier, and describing (20) like the form of (9) , we have the following nonlocal evolution equation which is expected to approximate the solution of the original discrete model (20):

$$\begin{aligned} \left\{ \begin{aligned} u_{t}^\varepsilon&= d_u K*u^\varepsilon -\frac{ 2d_u u^\varepsilon }{l^2} + g_1( u^\varepsilon , v^\varepsilon ),\\ v_{t}^\varepsilon&= d_v K*v^\varepsilon -\frac{ 2d_v v^\varepsilon }{ l^2 } + g_2( u^\varepsilon , v^\varepsilon ), \end{aligned} \right. \quad \text {in } {\mathbb {T}}\times \{t>0\}, \end{aligned}$$

where $$K = (\rho _{\varepsilon , -l} + \rho _{\varepsilon , l})/ l^2 $$.

#### Delta–Notch interaction system

Secondary, we consider the continuation method to the general Delta–Notch interaction system. Let $D_i=D_i(t)$ and $N_i=N_i(t)$ be the expression of Delta, and Notch signal in the cell $c_i, \ (i=1, \ldots , N)$, respectively. The simple description for the Delta–Notch signaling in the framework of the discrete model is given by the following system Collier et al. (1996):

22 $$\begin{aligned} \left\{ \begin{aligned} N_{i,t}&= f ( D_{i-1}, D_{i+1} ) + g_1(N_i, D_i),\\ D_{i,t}&= g_2(N_i, D_i), \end{aligned} \right. \quad t>0, \quad i=1, \ldots , M, \end{aligned}$$

where *f* is the function $f_{ \text {lat} }$ defined in (4) or the function depending $f_{ \text {lat} }$, $g_1$ and $g_2$ are the functions for reactions in this sub-subsection, and we replace the literature for the cell number *N* with $M \in {\mathbb {N}}$ for the clear description in this sub-subsection. Similarly to Sect. 2.1, the changing the variables in Eq. (22) as

$$\begin{aligned} N(x,t) : = \sum _{i=1 }^{M } N_{i}(t) \chi _{c_i}(x), \quad D(x,t) : = \sum _{i=1 }^{M } D_{i}(t) \chi _{c_i}(x) \end{aligned}$$

yield the following equation

23 $$\begin{aligned} \left\{ \begin{aligned} N_{t}&= f( D(x-l,t), \ D(x+l,t) ) + g_1(N, D),\\ D_{t}&= g_2(N, D), \end{aligned} \right. \quad \text {in} \ {\mathbb {T}}\times \{t>0\}. \end{aligned}$$

Indeed, if the initial conditions of (22) and (23) are the same, the solutions of (22) and (23) are equivalent for any time $t>0$. Regarding the shift operator as the convolution with the Dirac Delta function, we approximate it by the convolution with the mollifier. If *f* is linear such as $f_{ \text {lat} }$, we have the following nonlocal evolution equation similarly to (9):

$$\begin{aligned} \left\{ \begin{aligned} N_{t}^\varepsilon&= K*D^\varepsilon + g_1(N^\varepsilon , D^\varepsilon ),\\ D_{t}^\varepsilon&= g_2(N^\varepsilon , D^\varepsilon ), \end{aligned} \right. \quad \text {in} \ {\mathbb {T}}\times \{t>0\}, \end{aligned}$$

where $K = (\rho _{\varepsilon , -l} + \rho _{\varepsilon , l})/ {2}$. Even if the number of the unknown variables is increased, our method is applicable to make the discrete model continuous.

### Two-dimensional space

In this subsection we will explain our continuous method for the discrete model in the two-dimensional case. As the continuation method for the scalar equation can be applied to the system similarly to one-dimensional case, we firstly deal with the scalar discrete model in two-dimensional space. As considering the model in two-dimensional case, the number of terms in the intercellular interaction is increased. Accordingly, the number of the terms of shift or convolution with mollifier is increased in the continuation method. The procedure of continuous method for the reaction term is the same as the explanation of the previous subsections.

We set the square region, and impose the periodic boundary condition in this subsection.

#### Square lattice

We perform the continuation method for the discrete model in the square lattice. This mathematical model corresponds to the situation that square cells are packed in a plane in the development of multicellular organs. Dividing the square region into $N^2$ square parts of lattices, we label each lattice as $c_{i,j}, \ (i,j=1, \ldots , N)$, and denote the horizontal and vertical length of a lattice by $l_x>0$ and $l_y>0$, respectively. Thus, the region is described by

$$\begin{aligned} {\mathbb {T}}^2:=[0, Nl_x]\times [0, Nl_y]. \end{aligned}$$

One divided region corresponds to one cell or lattice as shown in Fig. 1.

For the simplicity, we suppose that region is a regular square. Then the scalar discrete model with intercellular interaction can be described as follows:

24 $$\begin{aligned} u_{i, j, t} = f( u_{i-1, j}, \ u_{i+1, j}, \ u_{i, j}, \ u_{i, j-1}, \ u_{i, j+1} )+g( u_{i, j}), \quad t>0, \quad i,j=1, \ldots , N, \end{aligned}$$

where $u_{i,j}=u_{i,j}(t)$ is denoted by the concentration or density of some substances on $c_{i,j}$ at time $t>0$, $f:{\mathbb {R}}^5 \rightarrow {\mathbb {R}}$ and $g:{\mathbb {R}}\rightarrow {\mathbb {R}}$ are intercellular and reaction functions, respectively, in this sub-subsection. If *f* is a linear function, it can be generally written by

$$\begin{aligned} f(u_{-2}, u_{-1}, u_{0}, u_{1}, u_{2} )= \sum _{i=-2}^{2}a_{i} u_{i} \end{aligned}$$

with $\{ a_i \}_{i=-2}^2 {\subset \ } {\mathbb {R}}$. The typical examples of *f* are diffusion and lateral inhibition as follows:

$$\begin{aligned}&f_{\varDelta }( u_{i-1, j}, u_{i+1, j }, u_{i,j}, u_{i, j-1}, u_{i, j+1} ) = \frac{ u_{i+1, j} -2 u_{i,j} + u_{i-1, j } }{ l_x^2 } + \frac{ u_{i, j+1} -2 u_{i,j} + u_{i, j-1} }{ l_y^2 }, \\&f_{\varDelta \times }( u_{i-1, j-1}, u_{i+1, j-1 }, u_{i,j}, u_{i-1, j-1}, u_{i+1, j+1} )\\&\quad = \frac{ u_{i+1, j+1} -2 u_{i,j} + u_{i-1, j-1 } }{ l_x^2 } + \frac{ u_{i+1, j+1} -2 u_{i,j} + u_{i-1, j-1} }{ l_y^2 }, \\&f_{\text {lat}}(u_{i-1, j}, u_{i+1, j }, u_{i, j-1}, u_{i, j+1} ) = \frac{ - u_{i+1, j} - u_{i-1, j} - u_{i, j+1} - u_{i, j-1} }{4}, \end{aligned}$$

where $f_{\varDelta \times }$ is referred from Chow (2003). For this discrete model we prepare the following characteristic function at position $(x,y) \in {\mathbb {T}}^2$:

$$\begin{aligned} \chi _{c_{i, j}}(x, y)= \left\{ \begin{array}{ll} 1 &{}\quad \text {if} \ (x,y) \in c_{i,j},\\ 0 &{}\quad \text {otherwise}. \end{array} \right. \end{aligned}$$

For the Eq. (24) for $i,j=1, \ldots , N$, we change the variables similarly to Sect. 2.1 by using the characteristic function. Here setting the variable on ${\mathbb {T}}^2$ as

25 $$\begin{aligned} u(x,y,t) := \sum _{i,j=1}^N u_{i,j}(t) \chi _{c_{i,j}}(x,y ), \end{aligned}$$

we have

$$\begin{aligned} u_{t}= f ( u(x-l_x,y,t), \ u(x+l_x,y,t), \ u(x,y,t), u(x, y-l_y, t), \ u(x,y+l_y, t) )+g( u ) \end{aligned}$$

from same calculation as that on one-dimensional case. We put the specific calculation in “Appendix C”. The discrete model is successfully converted into the continuous model. Similarly to the case in one dimension, approximating the shift operator by the convolution with the mollifier yields the nonlocal evolution equation:

$$\begin{aligned} u_{t}^\varepsilon&= f ( (\tau _{l_x,0}{\varvec{\rho }}_\varepsilon ) * u^\varepsilon , \ (\tau _{-l_x,0}{\varvec{\rho }}_\varepsilon ) * u^\varepsilon , \ u^\varepsilon , \ (\tau _{0,l_y}{\varvec{\rho }}_\varepsilon ) * u^\varepsilon , \ (\tau _{0, -l_y}{\varvec{\rho }}_\varepsilon ) * u^\varepsilon )+g( u^\varepsilon ), \end{aligned}$$

where we define the shift operator $\tau _{l,m}$ as

$$\begin{aligned} \tau _{l,m} u = u(x-l, y-m). \end{aligned}$$

If *f* is linear, the description with the kernel is given as follows

$$\begin{aligned} u_{t}^\varepsilon = K * u^\varepsilon + a_0 u^\varepsilon +g( u^\varepsilon ), \end{aligned}$$

where

26 $$\begin{aligned} K= a_{-2} (\tau _{l_x,0}{\varvec{\rho }}_\varepsilon )+ a_{-1} (\tau _{-l_x,0}{\varvec{\rho }}_\varepsilon ) + a_1 (\tau _{0,l_y}{\varvec{\rho }}_\varepsilon ) + a_2 (\tau _{0,-l_y}{\varvec{\rho }}_\varepsilon ). \end{aligned}$$

**Fig. 5:**
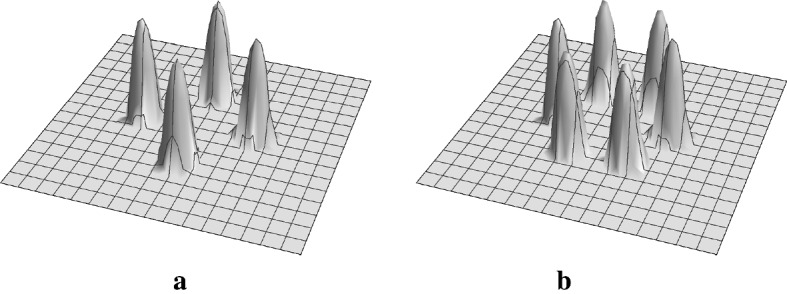
The profile of the kernel of the intercellular interaction (26) with $a_j=1, \ (j=-2,-1,1,2)$ and (29) with $a_j=1, \ (j=1, \ldots , 6)$ on the square **a** and hexagonal **b** lattices, respectively, and $\varepsilon =0.7$

As mentioned above, our method enables us to derive the continuous model and nonlocal evolution equation for the original discrete model. The profile of the kernel for $f_{\varDelta }$ on the square lattice is shown in Fig. 5a.

#### Hexagonal lattice

In this sub-subsection, we explain the continuation method on hexagonal lattice. Due to the hexagonal lattice, the direction of the shift operator is different from that in the square lattice. Dividing the region into the regular hexagons, we label each mesh as $c_j, \ j=1,\ldots , N$ as in Fig. 1b. In this sub-subsection we use the index of *j* for the label of each cell instead of *i*. We write the neighboring cells around the cell $c_j$ as $c_{\varLambda _j^k}, \ k=1,\ldots , 6$, i.e., $\Lambda _j^k \ (k=1, cdots, 6)$ is the index of neighboring cells for the $j\hbox {th}$ cell.

The typical discrete model on the hexagonal lattice can be described as follows:

$$\begin{aligned} u_{j, t} = f( u_{\varLambda _j^1}, \ u_{\varLambda _j^2}, \ u_{\varLambda _j^3}, \ u_{\varLambda _j^4}, \ u_{\varLambda _j^5}, \ u_{\varLambda _j^6}, u_j )+g( u_{j}), \quad t>0, \quad j=1, \ldots , N, \end{aligned}$$

where $f:{\mathbb {R}}^7 \rightarrow {\mathbb {R}}$ and $g:{\mathbb {R}}\rightarrow {\mathbb {R}}$ are intercellular and reaction functions, respectively, here. The linear intercellular interaction *f* on the cell $c_i$ is generally given by

$$\begin{aligned} f(u_{\varLambda _j^1}, \ u_{\varLambda _j^2}, \ u_{\varLambda _j^3}, \ u_{\varLambda _j^4}, \ u_{\varLambda _j^5}, \ u_{\varLambda _j^6}, u_j)= \sum _{k=1}^6 a_k u_{ \varLambda _j^k } + a_0 u_{ j }. \end{aligned}$$

Similar to the previous sub-subsection, the typical examples of *f* are diffusion and Delta–Notch interaction as follows:

$$\begin{aligned} f_{\varDelta }(u_{\varLambda _j^1}, \ u_{\varLambda _j^2}, \ u_{\varLambda _j^3}, \ u_{\varLambda _j^4}, \ u_{\varLambda _j^5}, \ u_{\varLambda _j^6}, u_j )&= \frac{ u_{\varLambda _j^1}+ u_{\varLambda _j^2} + u_{\varLambda _j^3} + u_{\varLambda _j^4} + u_{\varLambda _j^5} + u_{\varLambda _j^6} -6 u_j }{ l^2 }, \\ f_{\text {lat}}( u_{\varLambda _j^1}, \ u_{\varLambda _j^2}, \ u_{\varLambda _j^3}, \ u_{\varLambda _j^4}, \ u_{\varLambda _j^5}, \ u_{\varLambda _j^6})&= \frac{-u_{\varLambda _j^1} - u_{\varLambda _j^2} - u_{\varLambda _j^3} -u_{\varLambda _j^4} - u_{\varLambda _j^5} - u_{\varLambda _j^6} }{ {6} }. \end{aligned}$$

Utilizing the characteristic function $\chi _{c_j}(x,y)$, we define the variable at position $(x,y) \in {\mathbb {T}}^2$ at time $t>0$ as

27 $$\begin{aligned} u(x,y,t) := \sum _{ j=1}^N u_{ j }(t) \chi _{c_{j}}(x,y ). \end{aligned}$$

As the derivation is similar to those in previous sections, we put detailed calculations in “Appendix C”. Changing the variable through the characteristic function as in the previous Sect. 2.5.1, we obtain the continuous model:

$$\begin{aligned} \begin{aligned}&u_t = f\Big (u(x, y+l,t), u \Big (x+\frac{\sqrt{3}}{2}l, y + \frac{1}{2}l, t \Big ), u \Big ( x+\frac{\sqrt{3}}{2}l, y - \frac{1}{2}l, t \Big ), \\&\quad u(x, y-l,t), u\Big ( x-\frac{\sqrt{3}}{2}l, y - \frac{1}{2}l, t \Big ), u \Big (x-\frac{\sqrt{3}}{2}l, y + \frac{1}{2}l, t\Big ), u(x,y,t)\Big ) +g(u). \end{aligned} \end{aligned}$$

We define the shift operator as

28 $$\begin{aligned} \begin{aligned}&\tau _{\varLambda ^k} u = u\Big (x + \cos \Big (\frac{\pi }{2} - \frac{\pi (k-1) }{3} \Big ) l, \\&\quad y + \sin \Big (\frac{\pi }{2} - \frac{\pi (k-1) }{3}\Big ) l\Big ), \quad k=1,\ldots , 6. \end{aligned} \end{aligned}$$

Approximating the shift operator by the convolution with the mollifier, we can derive the kernel corresponding to the intercellular interaction on the hexagonal lattice.

$$\begin{aligned} u_t^\varepsilon \nonumber&=K*u^\varepsilon +a_0 u^\varepsilon + g(u^\varepsilon ), \end{aligned}$$

where

29 $$\begin{aligned} K=K(x,y)=\sum _{k=1}^6 a_k (\tau _{\varLambda ^k} {\varvec{\rho }}_\varepsilon ) \end{aligned}$$

Figure 5b shows the profile of the kernel for the diffusion $f_{\varDelta }$ on the hexagonal lattice.

#### Isotropy of Delta–Notch signaling and radially symmetric kernel

In the previous sub-subsections we explained the continuation method for the uniform lattice with the uniform shape and size. However, the shape of the cells during development is not always uniform. Assuming the uniform lattice for the mathematical modeling for the phenomena might be artificial. Then we propose a kernel of our continuation method conserving the lattice size implicitly without assuming the uniform lattice based on the biological experiments.

**Fig. 6:**
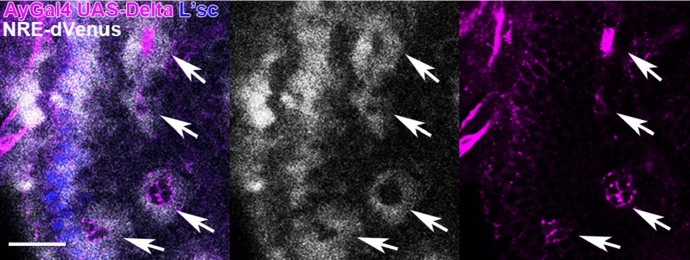
Notch activity as visualized by *NRE-dVenus* (white) is elevated in cells adjacent to the clones expressing *UAS-Delta* under the control of *AyGal4* (magenta) due to *trans*-activation, but is repressed in cells expressing *UAS-Delta* due to *cis*-inhibition (arrows). The wave front of the proneural wave is visualized by L’sc (blue). Scale bar, 20 $\mu \hbox {m}$

As explained in Sect. 1, the fly brain is often used for the study of neurogenesis mediated by the Delta–Notch signaling system. The shape of the NEs and neuroblasts (NBs), neural stem-like cells, in the fly brain looks various. Activation of Notch signaling is induced by binding of the Notch receptor with the Delta ligand expressed in adjacent cells at the cell surface. Therefore, activation of Notch signaling might be affected by the shape of the cell membrane. However, as various stochastic noise and other signaling pathway, other than Delta–Notch signaling are involved during development (Tanaka et al. 2018), we conjecture that the shape of the activated region of Notch may become isotropic and averaged. We asked how Notch signaling is activated when Delta is artificially expressed in a small number of cells. In this condition, the Notch activity was visualized by using the *NRE-dVenus* transgenic construct (Housden et al. 2012). As shown in Fig. 6, Notch signaling was activated in a group of cells immediately adjacent to the Delta-expressing cells through *trans*-activation forming a concentric distribution pattern. The inactivation of Notch signaling within the Delta expressing cells is most likely due to the effect of *cis*-inhibition (del Álamo et al. 2011; Sprinzak et al. 2010). This result suggests that the shape of cells do not affect the spatial activation pattern of Notch *in vivo*.

Based on this experimental results, we propose the following shape for the kernel

30 $$\begin{aligned} K(x,y)= \frac{1}{2 \pi l} \rho _\varepsilon \left( \sqrt{x^2+y^2} - l \right) . \end{aligned}$$

**Fig. 7:**
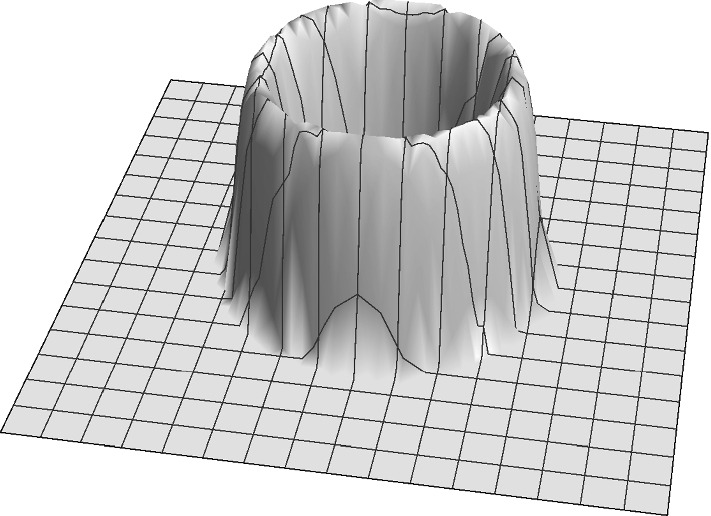
Profile of the radially symmetric kenrel (30) with $\varepsilon =0.7$

The profile of this kernel is shown in Fig. 7. The donut-like pattern of Notch activation is consistent with the concentric shape of the kernel used in (30).

By using this shape of kernel, the nonlocal operator becomes radially symmetric. This radially symmetric kernel is also applicable to describe the signaling system with projections of cells such as pigment cells in the skin of fishes Watanabe and Kondo (2015). As a result, the analysis of the continuous model from the discrete model becomes more available for the mathematical analysis.

## Applications

In this section we will apply our continuation method for some discrete models of previous studies, and perform the numerical simulations to investigate how patterns are generated.

### Continuous model of the proneural wave

**Fig. 8:**
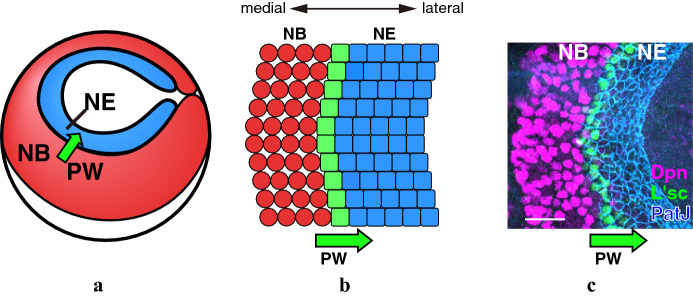
The propagation of the proneural wave. **a** Schematic depiction of the developing fly brain. **b** Schematic depiction of the proneural wave propagation. **c** A confocal image of the proneural wave propagation. NEs (labeled with PatJ, Blue), PW (labeled with L’sc, green), and NBs (labeled with Dpn, magenta) are shown. Scale bar, 20 $\mu \hbox {m}$

The developing fly brain looks like a hemisphere. During early stages of development, undifferentiated NEs proliferate and these NEs differentiate into NBs later stages of development. The transition from NEs to NBs propagates from the medial to the lateral directions. Since the transition is visualized by the transient expression of L’sc, which is one of the AS-C complex member and acts as a proneural factor, the propagation of the differentiation in the fly brain is called the *proneural wave* (PW) (Yasugi et al. 2008). In Fig. 8, NEs, NBs, and the PW are visualized by the staining for PatJ (blue), Deadpan (magenta), and L’sc (green), respectively. The propagation of the PW is regulated by the interaction of EGF and Delta–Notch pathways (Yasugi et al. 2010). In Sato et al. (2016), a discrete model with four factors, EGF, Notch, Delta, and AS-C has been proposed to investigate how Delta–Notch signaling controls the PW propagation. For the simplicity the mathematical model for the PW was proposed by dividing the region to uniform square or hexagonal lattice, and each lattice is labeled $i, j\hbox {th}$ cells as $c_{i, j}$ in Sato et al. (2016), Tanaka et al. (2018). The dynamics of Notch, Delta, and AS-C are given by the discrete model, and the dynamics of EGF is given by the continuous model at time $t > 0$ as follows:

31 $$\begin{aligned} \left\{ \begin{array}{l l l} \dfrac{\partial E}{\partial t}&{}=&{} d_e \varDelta E - k_e E + a_eA(A_0 - A), \ \\ \dfrac{d N_{i,j}}{d t} &{}=&{} - k_n N_{i,j} + d_t \displaystyle {\sum _{l,m \in \varLambda _{i,j}}} D_{l,m} - d_c N_{i,j} D_{i,j},\\ \dfrac{d D_{i,j}}{d t} &{}=&{} - k_d D_{i,j} + a_d A_{i,j} (A_0-A_{i,j}), \\ \dfrac{d A_{i,j}}{d t} &{}=&{} e_a(A_0-A_{i,j})\max \{ E_{i,j}-N_{i,j}, 0\}, \end{array} \right. \text {in} \ \varOmega \times \{ t>0\}, \end{aligned}$$

where the calculation region is set as $$\varOmega =[0,L_x]\times [0,L_y], \ L_x, L_y>0$$, $$E=E(x, y, t)$$ is denoted by the composite variable for the EGF ligand concentration and EGF signaling at position *x* and time $$t>0$$, $$N_{i,j}=N_{i,j}(t)$$, and $$D_{i,j}=D_{i,j}(t)$$ are variables for the Notch signal activity and Delta expression in the $$i\hbox {th}$$ and $$j\hbox {th}$$ cells at time $$t>0$$, respectively, $$A_{i,j}=A_{i,j}(t)$$ is a variable for the level of the differentiation reflecting the expression of AS-C in the $$i\hbox {th}$$ and $$j\hbox {th}$$ cells at time *t*, and $$d_e, k_e, a_e$$, $$ k_n, d_t, d_c, k_d, a_d$$ and $$e_a$$ are positive constants. The variables without index is the unknown variables which are extended the whole region by the characteristic function, and $$E_{i,j}$$ is the average value of EGF in the $$i,j\hbox {th}$$ cell $$c_{i,j}$$. As Notch signaling in the $$c_{i,j}$$ cell is activated by Delta in the neighboring cells which surround the $$c_{i,j}$$ cell, the sum of the expression of Delta in the neighboring cells $$D_{l,m}(t)$$ is imposed in the second equation. As considering the discrete model, the concentration of Delta, Notch and AS-C is uniform in each cell $$c_{i,j}$$. The information of the cell membrane is not included in this model. It has been reported that this discrete model replicates the mode of the PW with the suitable parameter from the numerical simulations (Sato et al. 2016; Tanaka et al. 2018). Moreover, as increasing the strength of the lateral inhibition in the parameter, (31) could reproduce the nonuniform propagation, called the salt-and-pepper patterns as introduced in Sect. 1, in the numerical simulation, thereby indicating the existence of the salt-and-pepper pattern in the fly brain.

To apply the analytical theory, for example the theory of the traveling wave solution, or reduction method, we perform the continuation method to this model (31). For this model, we assume the following:

(A5) There are spatial distributions of Delta, Notch and AS-C in the cells.
(A6) The trigger of the differentiation at each point in the cells is determined by the value of EGF instead of the value of the integration of EGF in a cell.

From the assumption (A5), using the characteristic function, we change the variable as follows:

$$\begin{aligned} D(x,y,t) :=&\sum _{i,j=1}^N D_{i,j}(t) \chi _{c_{i,j}}(x,y ), \quad N(x,y,t) := \sum _{i,j=1}^N N_{i,j}(t) \chi _{c_{i,j}}(x,y ), \\ A(x,y,t) :=&\sum _{i,j=1}^N A_{i,j}(t) \chi _{c_{i,j}}(x,y ). \end{aligned}$$

We propose the following the continuous model:

32 $$\begin{aligned} \left\{ \begin{array}{l l l} \dfrac{\partial E}{\partial t}&{}=&{} d_e \varDelta E - k_e E + a_eA(A_0 - A), \ \\ \dfrac{\partial N}{\partial t} &{}=&{} - k_n N + d_t K*D - d_c N D,\\ \dfrac{\partial D}{\partial t} &{}=&{} - k_d D + a_d A (A_0-A), \\ \dfrac{\partial A}{\partial t} &{}=&{} e_a(A_0-A)\max \{ E-N, 0\}, \end{array} \right. \text {in} \ \varOmega \times \{ t>0\}. \end{aligned}$$

where the profile of the kernel $K=K(x,y)$ is determined by the profile of the lattice. For the simple description, we impose the local term of the EGF in the $\max$ function of the fourth equation based on the assumption (A6).

**Fig. 9:**
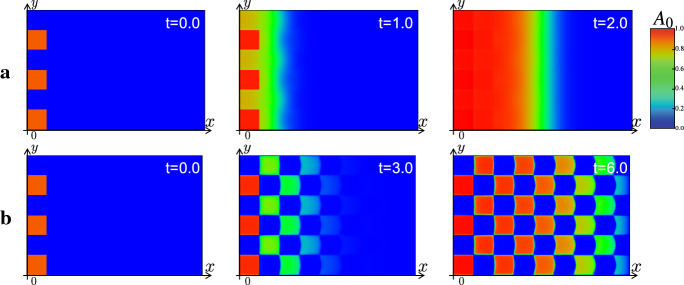
The results of the numerical simulations for the state of the differentiation *A* in the continuous model (32) with the kernel corresponding to the square lattice. The parameters are $d_e=2.0, k_e=1.0, k_n=3.0, d_t=2.0, d_c=0.5, k_d=1.5, a_d=1.0, A_0=1.0, e_a=10.0, \varepsilon =0.2$, $l=1$ and $K=\sum _{j=1}^4 {\varvec{\rho }}_{\varepsilon }( x - l * e^{i \pi j /2 } )$ is used. The zero flux boundary, the Dirichlet boundary and the periodic boundary conditions are imposed in $\{ x=0 \}$, $\{ x=L_x \}$ and $\{ y=0\} \cup \{ y=L_y\}$, respectively. The high and low expression levels of *A* are shown in red and blue as indicated in the color bar, which is applied in the following Figs. 10, 11, 12, 13 and 14. **a** PW with $a_e=5.0$, **b** Salt-and-pepper pattern with $a_e=1.0$

We perform the numerical simulation to investigate whether the continuation method for the model (31) is effective or not. First, we perform the numerical simulations by using the kernel corresponding to Delta–Notch interaction on the square and hexagonal lattices. Figure 9 shows the numerical results of (32) with the kernel corresponding to square lattice. The uniform propagation of the differentiation corresponding to the PW is replicated with suitable parameters as in Fig. 9a. As the parameter corresponding to the strength of activation of EGF $a_e$ is decreased, the strength of the lateral inhibition by Delta–Notch becomes relatively larger. In this situation, the continuous model with the kernel reproduces the nonuniform propagation of the differentiation of AS-C corresponding to the salt-and-pepper pattern as in Fig. 9b. In this numerics, the square salt-and-pepper pattern does not depend on the numerical mesh in the code of numerical simulation. This one square region reflects the size and shape of one cell. We put the numerical results of the original discrete model for the PW reported in Sato et al. (2016), Tanaka et al. (2018) in “Appendix A”.

**Fig. 10:**
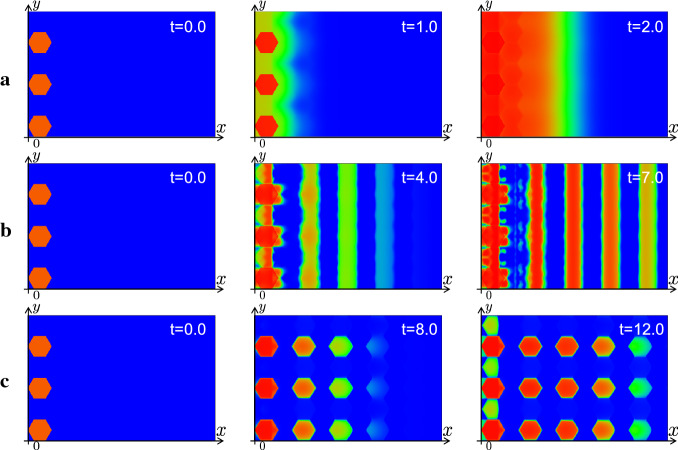
The results of the numerical simulations for the state of the differentiation *A* in the continuous model (32) with the kernel corresponding to the hexagonal lattice. The parameters are same as that in Fig. (9) and $K(x,y)=\sum _{k=1}^6 (\tau _{\varLambda ^k} {\varvec{\rho }}_\varepsilon )$ is used. **a** PW with $a_e=5.0$, **b** Stripe pattern with $a_e=2.1$, **c** Salt-and-pepper pattern with $a_e=1.0$

Secondary, the numerical results of the continuous model with the kernel corresponding to Delta–Notch interaction on hexagonal lattice are shown in Fig. 10. Similarly to Fig. 9, with the large value of strength of the activation for the EGF $a_e$ the continuous model with the kernel corresponding to the hexagonal mesh has replicated the mode of the PW. As decreasing the value of $a_e$, the stripe propagation of the differentiation is reproduced. Furthermore, by decreasing the value of $a_e$ more, the nonuniform propagation of the differentiation corresponding to the salt-and-pepper pattern has been reproduced. We can observe that each differentiated region exhibits the hexagonal shape. Even though the prepared mesh in the code of the numerical simulation is square, we can replicate the hexagonal patterns in the continuous model. This numerical results suggest that we can directly introduce the information of the spatial discreteness into the continuous model, and the solution of the continuous model with the suitable kernel can reproduce the solution of discrete model.

**Fig. 11:**
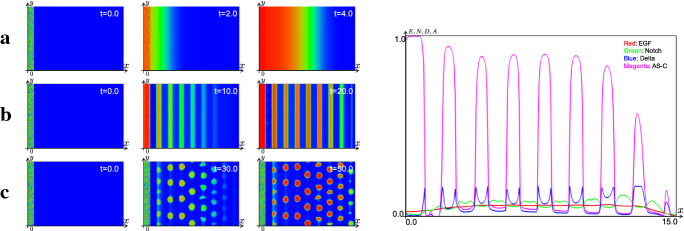
The results of the numerical simulations for the state of the differentiation *A* in the continuous model (32) with the kernel corresponding to the averaged cell (30). The parameters are same as that in Fig. 9, except for $k_n=10.0$, $d_c=5.0$, and $d_t=4\pi$ and (30) is used as the kernel. Left: **a** PW with $a_e=3.0$, **b** Stripe pattern with $a_e=0.7$, **c** Salt-and-pepper pattern with $a_e=0.4$. Right: Section of numerical simulation of **b** at $t=20.0$

Next, we perform the numerics with the radially symmetric kernel (30). As shown in Fig. 11, the mode of uniform PW and stripe propagation have been reproduced depending on the value of $$a_e$$. Moreover, as in Fig. 11c, the continuous model has replicated the propagation of the salt-and-pepper pattern even by using the radially symmetric kernel (30). As shown in Fig. 11c, the profile of the differentiated region is spotted and can be interpreted that it indicates the shape of the averaged cells. Although we also use square mesh in the numerical simulation code in these numerics, the continuous model with radially symmetric kernel can reproduce the uniform and nonuniform propagations. The right side of Fig. 11 shows the section of the numerics of (b) in $$y=L_y/2 $$ at $$t=20.0$$. The blue curve corresponds to the profile of Delta, and we can observe that the Delta is expressed at the wave front. These numerical results are consistent with the observation that Delta expression is localized to the cell membrane in the real fly brain. In the view of mathematical modeling, it is explained that the term of the activation from the AS-C in the front $$a_dA(A_0-A)$$ is imposed in the third equation of (32). We succeeded in reproducing the realistic pattern through our continuation method.

As mentioned above, the results of our numerics suggest that we can analyze the solution observed in the discrete model in the framework of the continuous model equipping the spatially discretized initial data. In the successive subsections, we perform the numerical simulations of discrete model of the PW on growing domains and expansions of the model on the sphere by using our continuation method to investigate the realistic situation of the developing fly brain.

### Modeling of cell division on the discrete model

Owing to our continuation method by the convolution with the kernel, we are able to model the cell division and proliferation in the discrete model. In this subsection, we explain this application by using the model of the PW.

During the process of the PW, the nonuniform cell division occurs on the surface. The fly brain develops via an early NE expansion phase followed by a differentiation phase from NEs to NBs (Egger et al. 2007; Hofbauer and Campos-Ortega 1990). When we try to add the effect of cell division to the discrete model, it is often artificial because we must decide the timing, direction, and number of cell division. However, we can introduce this effect naturally in our continuation method by expressing it as the domain growth. We put the explanations of the basic idea for the domain growth in “Appendix D”. Using the method of the domain growth, we add the effect of cell division to (31) as follows:

33 $$\begin{aligned} \left\{ \begin{array}{l} \dfrac{\partial E}{\partial t}=\dfrac{d_e}{\varGamma _y}\dfrac{\partial }{\partial y}\left( \dfrac{1}{\varGamma _y} \dfrac{\partial E}{\partial y}\right) -k_eE+a_eA(A_0-A)-\eta E, \\ \dfrac{\partial N}{\partial t}=-k_nN+d_t{\tilde{K}}{*}D-d_cND-\eta N, \\ \dfrac{\partial D}{\partial t}=-k_dD+a_dA(A_0-A)-\eta D ,\\ \dfrac{\partial A}{\partial t}=e_a(A_0-A)\max \{E-N,0\}-\eta A, \\ \dfrac{\partial \varGamma _y}{\partial t}=\eta \varGamma _y, \end{array} \right. \ \ {\mathrm {in}}\ (0,L(0)) \times \{t>0\},\nonumber \\ \end{aligned}$$

where $K(x)=\rho _{\varepsilon }(x-l)+\rho _{\varepsilon }(x+l)$, ${\tilde{K}}$ is the kernel with changed variable of *K*, $\varGamma _y$ is a derivative bijection function, and $\eta$ is determined below. The detail of ${\tilde{K}}$ and $\varGamma _y$ are explained in “Appendix D”. Since AS-C can be regarded as the level of differentiation, we suppose that the cell is the NE if the value of *A*(*x*, *t*) is close to 0, and the cell is the NB if the value of *A*(*x*, *t*) is close to 1. To express the nonuniform cell division of the PW, $\eta =\eta (y,t,A)$ is given by the monotone decreasing function with respect to *A*, because NE is divided on the surface of fly brain. Here, we assume that the point satisfying $A(t,x)\ge A^*$ is not divided on the surface. Now, we set

34 $$\begin{aligned} \eta (y,t,A)= {\left\{ \begin{array}{ll} \eta _0 \left( 1-\dfrac{A(y,t)}{A^*}\right) , &{}\quad if\ A(y,t)\le A^{*},\\ 0, &{}\quad otherwise, \end{array}\right. } \end{aligned}$$

where $\eta _0$ is a constant.

**Fig. 12:**
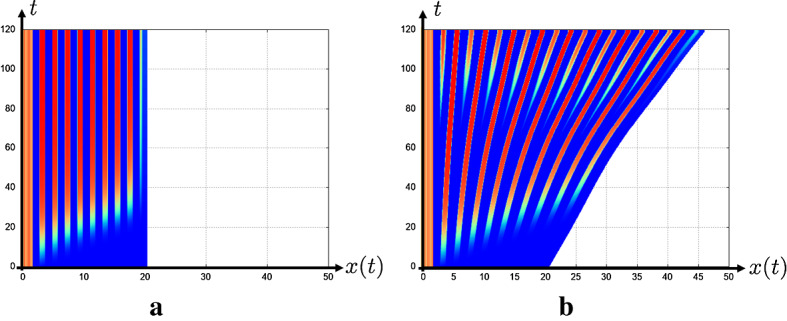
The results of the numerical simulations for the state of the differentiation *A* in the continuous model on growing domain (33). The parameters are same as that in Fig. 11, except for $d_t=4.0$, $a_e=0.1$ and $L(0)=20.0$. **a** No division with $\eta (y,t,A)\equiv 0$. **b** Nonuniform division using $\eta (y,t,A)$ defined (34) with $A^*=0.5$ and $\eta _0=0.01$

Figure 12 shows the numerical results of (33) in the cases of fixed domain and the nonuniform cell division, respectively. In the beginning of the numerical simulations, we observed the similar patterns in the both (a) and (b). However, the NBs newly appear between the valleys of regions of NBs in Fig. 12b as the time passes. This numerical result can be explained in the view of mathematical modeling as follows. The differentiation of *A* at each point is inhibited by *N* in the $\max$ function of the fourth equation in (33). *N* in a cell is activated by *D* which is activated at the wave fronts of the regions corresponding to the adjacent cells. Therefore, when the region corresponding to the NEs is close to the wave front, the differentiation is inhibited. Conversely, farther from the wave front the region corresponding to the NEs is, the weaker the strength of the lateral inhibition of Notch becomes. As the EGF diffuses to the region, the differentiation of NBs occurs in the valleys of the regions corresponding to NBs. At present, we consider the continuous model of the PW in one-dimensional space. In the future, we will try to calculate in two-dimensional space and on the sphere surface in order to apply the experiment. Furthermore, in Kawamori et al. (2011), it is reported that the wave front of the PW is dented in the clone of fly brain due to the fast NE’s division. Thus, we want to understand the reasons why the profile of the wave front is affected by the speed of the NE’s division from the viewpoint of the mathematical model.

### Description of discrete model on sphere surface

For another example of applications of our continuation method to mathematical modeling, we explain the description of the discrete model on sphere surface. We show that we can deal with the discrete model on the sphere surface by using the radially symmetric kernel (30).

Various pattern formations often occur in the region of sphere surface in the development of multicellular organisms. In the case of the PW investigated in the previous subsections, the fly brain is the hemisphere-like shape, and the PW sweeps across the surface. It is natural to construct the discrete model for the PW on the sphere surface, but the mathematical studies of the PW have been discussed on the 2D plane due to the technical difficulties of the discreteness in the numerical simulations on the sphere. Here, our continuation method can overcome these difficulties and enables us to deal with the model on the sphere surface as the continuous model equation. In practice, by applying the continuation method with the radially symmetric kernel with a radius $r>0$ in Sect. 2.5.3, we can compute the continuous model (32) on the sphere surface by the spectrum method. We put the explanations of the basic idea for the spectrum method on sphere surface in “Appendix E”. Using the spectrum method, we can compute the following model of the PW on the sphere surface numerically:

35 $$\begin{aligned} \left\{ \begin{array}{l} \dfrac{\partial E}{\partial t}=d_e\varDelta _{r{\mathbb {S}}^2} E-k_eE+a_eA(A_0-A), \\ \dfrac{\partial N}{\partial t}=-k_nN+d_tK*_{r{\mathbb {S}}^2}D-d_cND, \end{array} \right. \ \ {\mathrm {in}}\ r{\mathbb {S}}^2 \times \{t>0\}, \end{aligned}$$

where the equation of *D* and *A* are same as the equation (31), and $r{\mathbb {S}}^2$ is a sphere with radius $r>0$ and $K: [0, 2r]\rightarrow {\mathbb {R}}$ is defined as

$$\begin{aligned} K(x):=\rho _{\varepsilon }(x-l), \quad x \in [0, 2r]. \end{aligned}$$

The Laplace-Beltrami operator $\varDelta _{r{\mathbb {S}}^2}$ on the general sphere with the radius $r>0$ is given by

$$\begin{aligned} \varDelta _{r{\mathbb {S}}^2}&=\frac{1}{r^2}\varDelta _{{\mathbb {S}}^2}, \end{aligned}$$

where the definition of the Laplace–Beltrami operator is in “Appendix E”. The convolution operator on the sphere $*_{r{\mathbb {S}}^2}$ is computed as

$$\begin{aligned} K*_{r{\mathbb {S}}^2}u(x)&:=\int _{r{\mathbb {S}}^2}K(|x-y|)u(y)d\varOmega _{r}(y), \\&=r^2 \int _{{\mathbb {S}}^2}K_r(|x-y|)u_r(y)d\varOmega (y), \\&=K_r *_{{\mathbb {S}}^2} u_r(x), \end{aligned}$$

where

36 $$\begin{aligned} K_r(|\cdot |):=K(r|\cdot |),\ u_r(x):=u(r x)\ (x\in {\mathbb {S}}^2), \end{aligned}$$

$d\varOmega _{r}$ is denoted by the standard measure on $r{\mathbb {S}}^2$, and $*_{{\mathbb {S}}^2}$ is the convolution on the unit sphere. According to this calculation, we can rewrite the equation (35) on the unit sphere as follows:

37 $$\begin{aligned} \left\{ \begin{array}{l} \dfrac{\partial E_r}{\partial t}=\dfrac{d_e}{r^2}\varDelta _{{\mathbb {S}}^2} E_r-k_eE_r+a_eA_r(A_0-A_r), \\ \dfrac{\partial N_r}{\partial t}=-k_nN_r+d_t r^2 K_r*_{{\mathbb {S}}^2}D_r-d_cN_rD_r, \\ \dfrac{\partial D_r}{\partial t}=-k_dD_r+a_dA_r(A_0-A_r) ,\\ \dfrac{\partial A_r}{\partial t}=e_a(A_0-A_r)\max \{E_r-N_r-k_{in} J_r,0\}, \end{array} \right. \ \ {\mathrm {in}}\quad {\mathbb {S}}^2\times \{t>0\}, \end{aligned}$$

where $k_{in}$ is a positive constant, $J=J(x,t)$ reproduces the profile of the JAK/STAT signaling, which cancels the biological noise in the fly brain reported in Tanaka et al. (2018), and the notation of unknown variables are based on (36). When we calculate the equation (37) by the spectrum method, the spatial noise arises from finite spherical harmonic expansion. Therefore, we need the effect of JAK/STAT as the role of the noise reduction (Tanaka et al. 2018). For simplicity, we assume that the value of *J*(*x*, *t*) is spatially uniform. Furthermore, as the spatial interaction of the equation (37) are the diffusion term in first equation and the convolution term in second equation, we calculate numerically the evolution of *E* and *N* by the spectrum method and compute the evolution of *D* and *A* by the Euler method.

**Fig. 13:**
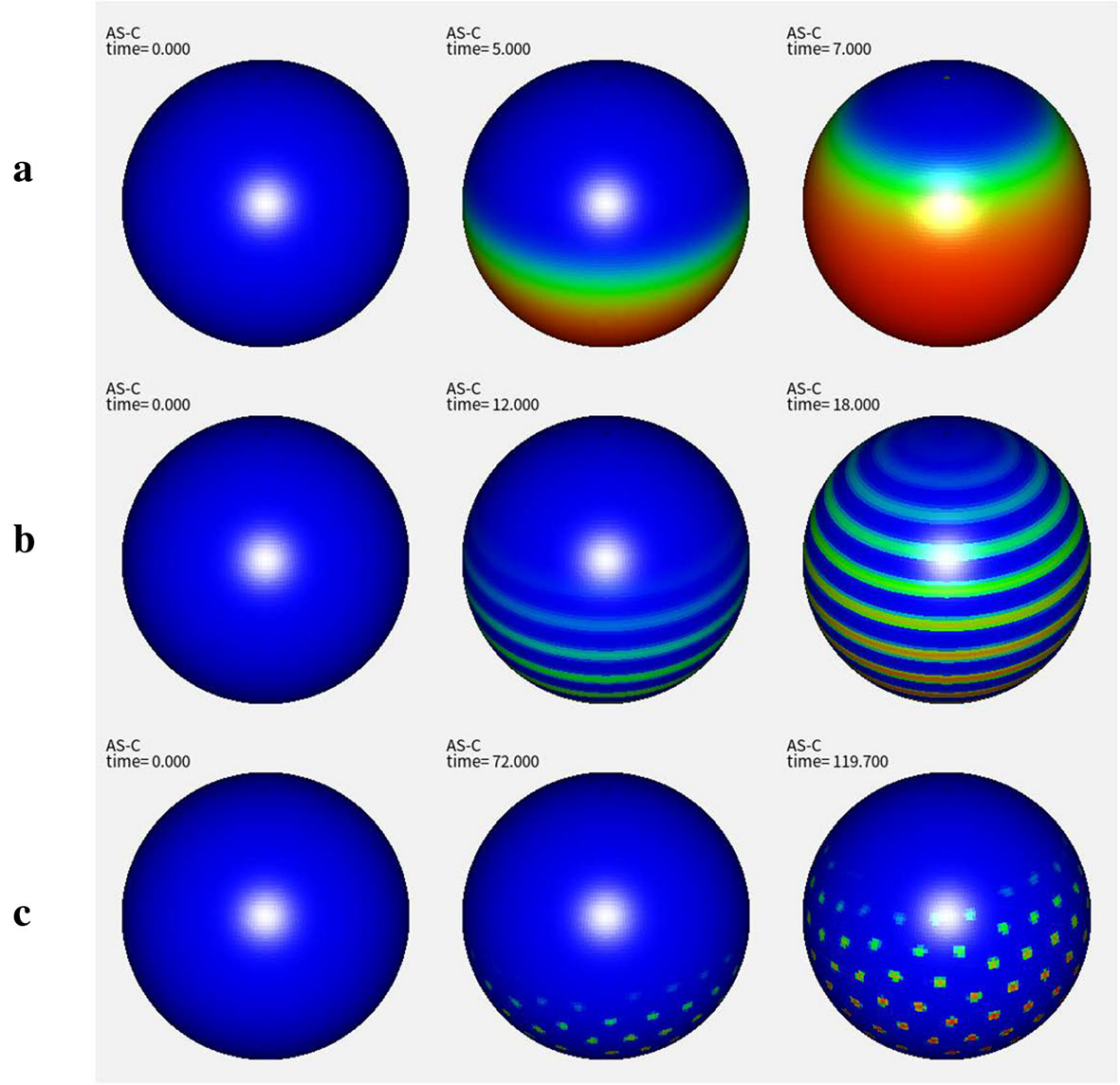
The results of the numerical simulations for the state of the differentiation *A* in the continuous model on the sphere surface (37). The parameters are same as that in Fig. 11, and $r=10.0$ and $k_{in}J\equiv 1.0\times 10^{-3}$. **a** PW with $a_e=2.0$. **b** Stripe pattern with $a_e=0.7$. **c** Salt-and-Pepper pattern with $a_e=0.4$

In numerical simulation of Fig. 13 at which parameters are the same as those of Fig. 11, we obtain numerical results of the propagation of AS-C similar to the case of the 2D plane when $r=10.0$. We can interpret the reason of this result as follows. When the radius of the sphere *r* is relatively large compared to the cell size, the curvature of the cell surface becomes small. From this, the cell on the sphere surface can be regarded as the same state as the case of the plane. Therefore, we obtain similar numerical results to those of Fig. 11.

**Fig. 14:**
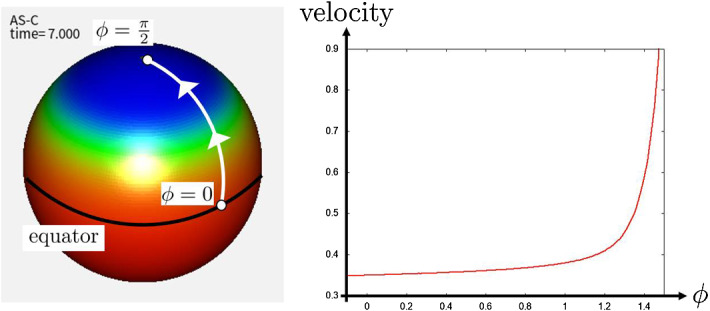
The velocity of PW for each position, when $d_t=0$ and other parameters are the same as that in Fig. 11. $\phi$ represents the latitude

We observe the PW is accelerated as the wave directs from the equator to the north pole as in Fig. 14. We think that this arises from the diffusion of the EGF ligand. Because the space become narrower as the wavefront approaches the pole, the EGF ligand accumulates and induce faster NB differentiation. It is not clear whether the speed of the PW progression is accelerated when the PW reaches close to the pole *in vivo*. This will be one of the interesting questions to be solved in the future by performing live imaging of the PW and quantitatively measured the speed of the wave progression.

### Application to the model of planar cellular polarity

In Ayukawa et al. (2014), a discrete model for planar cellular polarity (in short, PCP) of epithelial hair in the fly wing has been proposed by focusing on the interactions of transmembrane proteins, distal complexes and proximal complexes. The intercellular protein and the cytoplasmic component are asymmetry localized by the intercellular interactions. Due to the asymmetric localization of the proteins, the direction of an epithelial hair in a cell is determined. If the transmembrane receptor, Frizzled (Fz) is localized in a cell, the other transmembrane protein, Strabismus (Stbm), is localized in the opposite side of the cell membrane in the same cell. Each membrane protein interacts distal and proximal complexes in a cell, which causes the localization of the polarization of Fz and Stbm in the neighboring cells. Fz and Stbm in the neighboring cells are localized near sides. Localization of these proteins between adjacent cells leads to the local alignment of PCP among small group of cells.

A simple mathematical model succeeded in describing the mechanism of the PCP Ayukawa et al. (2014). In the modeling of Ayukawa et al. (2014), the region corresponding to the fly wing is divided into hexagonal lattices and each lattice is labeled as $c_i, \ i=1,\ldots , N$. By denoting the unknown variable for the direction of Fz in the $i\hbox {th}$ cell $c_i$ by $\theta _{i}=\theta _{i}(t)$ the following discrete model is proposed by the authors of Ayukawa et al. (2014):

38 $$\begin{aligned} \theta _{i,t} = \sum _{ j = 1}^6 \sin (\theta _{\varLambda _i^j} - \theta _i), \end{aligned}$$

where the number of the term in the linear combination with $\sin$ function depends on the spatial dimension and arrangement of the lattice. We perform our continuation method to this discrete model for PCP. Setting as

$$\begin{aligned} \theta (x,y,t)= \sum _{i=1}^N \theta _{i}(t) \chi _{c_{i}}(x,y ), \end{aligned}$$

we have the following continuous model by the changing the variables as in Sect. 2.5.2,

39 $$\begin{aligned} \theta _{t} = \sum _{j=1}^6 \sin (\tau _{\varLambda ^j} \theta - \theta ), \end{aligned}$$

where the shift operator is defined by (28). This model is equivalent to (38) if the initial data is equal. The form of the shift operator is changed depending on the shape of the set lattice.

We performed numerical simulations with the discretized initial datum on square lattice.

**Fig. 15:**
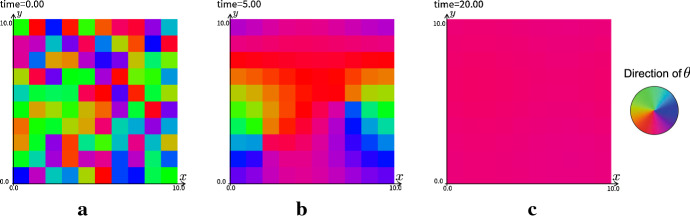
The numerical results of the continuous model for PCP with the discretized initial data corresponding to the discrete model on square lattice. The periodic boundary condition is imposed. The direction of $\theta _i$ in each cell $c_i$ is indicated by the color as in the color circle in right hand side. The mesh size of the numerics is $dx=0.02$, $l=1$, and the number of cell is equal to 100. **a** *t* = 0.00, **b** *t* = 5.00, **c** *t* = 20.0

**Fig. 16:**
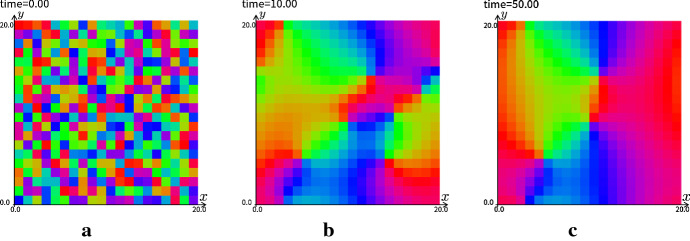
The numerical results of the continuous model for PCP with the discretized initial data corresponding to the discrete model on square lattice. The mesh size of the numerics is $dx=0.04$, $l=1$, and the number of cell is equal to 400. **a** *t* = 0.00, **b** *t* = 10.00, **c** *t* = 50.0

As in Fig. 15, we observe that the continuation model (39) with the discretized initial data can replicate the patterns in the discrete model (38). In this simulation, the color corresponding to the direction of an epithelial hair gets gradually uniform. This solution corresponds to that all epithelial hair grow in the same direction. On the other hand, as in Fig. 16, the solution corresponding to a swirl of the epithelial hairs is obtained in the steady-state as the number of cells is increased. This generation of the pole of $\theta$ is consistent to the report by Ayukawa et al. (2014).

In the discrete model, the number of the unknown variables is required for the same number of the cells. Accordingly, it is sometimes hard to compute the analytic calculations, for example, eigenvalue problem in the linear instability around the equilibrium solution. However, our method can reduce the discrete model with multiple components into scalar continuous model, and it gives us simpler calculations. We perform the linear instability of the model (38) by using the continuous model (39) in one-dimensional space. Suppose that the number of cells is *N*, and that the length is *l*. Setting the region as ${\mathbb {T}}= [0, Nl]$, we impose the periodic boundary condition. The model of PCP in one-dimensional space is given by the following form:

40 $$\begin{aligned} \theta _{t} = \sin (\theta (x-l,t) - \theta )+\sin (\theta (x+l,t) - \theta ), \quad \text {in} \ {\mathbb {T}}\times \{t>0\}. \end{aligned}$$

For this interval ${\mathbb {T}}$, we found that one equilibrium solution is given by $\theta ^* (x)= \sum _{j=1}^N \alpha _j \chi _{c_j}(x)$, where $\alpha _j = \frac{2 \pi }{N}j$. It is confirmed that

$$\begin{aligned}&\sin (\theta ^*(x-l) - \theta ^*)+\sin (\theta ^*(x+l) - \theta ^*) \\&\quad = \sin \left( \sum _{j=1}^N \chi _{c_j}(x)( \alpha _{j-1} - \alpha _j) \right) + \sin \left( \sum _{j=1}^N \chi _{c_j}(x)( \alpha _{j+1} - \alpha _j) \right) \\&\quad =\sin \left( -\frac{2 \pi }{N} \sum _{j=1}^N \chi _{c_j}(x) \right) + \sin \left( \frac{2 \pi }{N} \sum _{j=1}^N \chi _{c_j}(x) \right) \\&\quad = \sin \Big ( -\frac{2 \pi }{N} \Big ) + \sin \Big ( \frac{2 \pi }{N} \Big ) =0. \end{aligned}$$

Additionally, the constant solution $\theta ^*(x) = \alpha , \ \alpha \in [0, 2 \pi ]$ is also equilibrium solution of (40). The linear stability analysis around the equilibrium solutions is explained as follows. Letting the range of the linear operator be in ${\mathbb {R}}$, and setting the perturbation as $\theta (x,t) = \theta ^* + \varepsilon (x,t)$ and substituting the model (40) by it, we have

$$\begin{aligned} \varepsilon _t&= \sin \left( \sum _{j=1}^N \chi _{c_j}(x) ( \alpha _{j-1} - \alpha _j) +\varepsilon (x-l,t)- \varepsilon \right) \\&\quad + \sin \left( \sum _{j=1}^N \chi _{c_j}(x)( \alpha _{j+1} - \alpha _j) +\varepsilon (x+l,t)- \varepsilon \right) \\&= \sin \left( -\frac{2\pi }{N} +\varepsilon (x-l,t)- \varepsilon \right) + \sin \left( \frac{2\pi }{N} +\varepsilon (x+l,t)- \varepsilon \right) . \end{aligned}$$

Linearizing this problem around equilibrium solutions, we have the following eigenvalue problem:

41 $$\begin{aligned} \lambda \varphi&= \cos \Big ( \frac{2\pi }{N} \Big )\Big ( \varphi (x-l,t ) +\varphi (x+l,t) - 2\varphi \Big ) , \end{aligned}$$

where $\varphi = \varphi (x)$ is the eigenfunction associated by the eigenvalue $\lambda$. Plugging the $n\hbox {th}$ term of the Fourier series expansion

$$\begin{aligned} \varphi _n = a_n \exp \left( -\frac{ 2 n \pi i }{Nl}x\right) , \quad a_n:=\frac{1}{Nl} \int ^{Nl}_{0} \varphi (x) \exp \left( \dfrac{ 2 n \pi i }{Nl}x\right) dx \end{aligned}$$

to (41), where *i* is imaginary number, we obtain that

$$\begin{aligned} \lambda \varphi _n&= \cos \Big ( \frac{2\pi }{N} \Big ) \Big ( \exp \left( \frac{ 2 n \pi i }{N} \right) + \exp \left( -\frac{ 2 n \pi i }{N} \right) -2 \Big ) \varphi _n \\&= 2 \cos \Big ( \frac{2\pi }{N} \Big ) \Big (\cos \left( \frac{ 2 n \pi }{N} \right) -1 \Big ) \varphi _n. \end{aligned}$$

We have the eigenvalues $\lambda _n = 2 \cos \Big ( \frac{2\pi }{N} \Big ) \Big (\cos \left( \frac{ 2 n \pi }{N} \right) -1 \Big )$. The calculation of the eigenvalue of $f_{\varDelta }$ in the matrix form is also written in “Appendix B”, and each result is consistent. From this calculation, if the number of cell *N* is bigger than 3, we see that equilibrium solutions is linearly stable. By the same calculus, it is shown that the constant solution $\theta ^*$ is also linearly stable. Even in the two-dimensional case, our above method enables us to compute the linear stability analysis if we have the equilibrium solutions.

## Discussion

In this paper, we proposed a continuation method for discrete models, using shift and convolution operators while conserving the size and shape of cells and lattices. The proposed method enables the conversion of nonlinear discrete models into continuous models in a systematic manner, retaining the discreteness information. As the continuous model applied to our method with the shift operator is point-wisely equivalent to the original discrete model, the solutions are equal if the initial data are the same. The framework of the continuous model provides a few advantages, as per the analysis results. As in Sect. 3.4, we reproduced the pattern for the PCP in epithelial hair corresponding to that of the discrete model. Furthermore, we constructed the equilibrium solutions and performed the linear stability analysis in the continuous PCP model, using the Fourier series expansion. In Sect. 2.3, we showed that our continuation method can be applied to the discrete models on nonuniform lattices. Although the framework of discrete model is technically difficult to express the dynamics on nonuniform lattices, The proposed method enables us to treat the spatial non-uniformity on the continuous models mathematically. In the future, we will extend our work to the analyses and applications to this continuous models from the discrete models on the nonuniform lattices.

As a next step, we reduced the continuous model with the shift operators to the nonlocal evolution equation, using the approximation of the shift operator by convolution of a mollifier. We have also conducted the singular limit analysis of the discrete model and the nonlocal evolution equation in a one-dimensional interval with periodic boundary condition, showing that every solution is sufficiently close in $L^2({\mathbb {T}})$ space. This suggests that nonlocal evolution with suitable kernels is capable of approximating the solution of discrete models, if the initial data are the same. Using the nonlocal evolution equation with the kernel of mollifiers, we have succeeded in replicating the pattern observed in the original discrete PW model. When the intercellular interaction was linear, the profile of kernel was determined by the lattice shape as shown in (26) and (29). Using these kernels in the continuous model for the PW, we reproduced the square and hexagonal shapes of the salt-and-pepper patterns.

Furthermore, we experimentally confirmed the isotropy of the Delta–Notch signaling system in the real fly brain. Based on the biological experiment, we proposed a radially symmetric kernel for the domain comprising averaged cell shapes. Even with the radially symmetric kernel for the Delta–Notch signaling interaction, we could reproduce the various propagation patterns of the PW. The radially symmetric kernel can also be applied to the discrete models on nonuniform lattices if the molecular and cellular system is not affected by the shape of cells and lattice as explained in Sect. 2.5.3. For application to the biological experiments, using a kernel with a small width, such as the Friedrichs mollifier, yields results that are more compatible with experiments than the combination of the shift operators. However, the shift operator can be more convenient for the analysis. In Ei et al. (submitted), by arranging the Dirac Delta function radially as the kernel in the continuation method, the reduction method of the continuous model for PW into 1 or 2 variable system, and its numerical simulations are addressed. Nonlocal evolution equation with certain kernel is sometimes difficult to analyze. However, nonlocal evolution equation provides us with a unifying concept to mathematical modeling and analysis as it is capable of including partial differential equations such as the reaction diffusion systems and discrete models. Moreover, various analytical and numerical methods for partial differential equations are applicable to nonlocal evolution equations as explained in Sects. 3.2, 3.3 and 3.4. In future, we also intend to extend our work to other domains and include higher dimensions of singular limit analysis.

In Sect. 3.2, 3.3 and 3.4, we applied the continuous model on the PW progression and the PCP formation. We demonstrated that the continuous model can easily include the effect of NE cell proliferation and can expand the simulation result from the 2D plane to the 3D spherical surface. In fly brain development, the final numbers of NBs and neurons are dependent on the NE proliferation. Using biological experiments, it has been shown that NE expansion is regulated by several signaling pathways. The PI3K/Akt/TOR pathway promotes NE proliferation in a diet-dependent manner in the early stages of development (Franco and Carmena 2019; Lanet et al. 2013). The Hippo pathway inhibits the overgrowth of NEs and inactivation of Hippo signaling inhibits NB differentiation (Kawamori et al. 2011; Reddy et al. 2010; Richter et al. 2011). By combining biological experiments on these signaling pathways with numerical calculation, it will be possible to understand the *in vivo* situation of the PW progression in more detail. In this paper, we presented two biological examples, for which our numerical method is applicable. Here, we emphasize that the numerical method is also useful for other biological systems because our method is based on fundamental and conserved intercellular interactions. The continuation method and numerical calculation with continuous models will facilitate our understanding of a wide variety of biological processes, both quantitatively and qualitatively.

## Figures and Tables

**Fig. 17:**
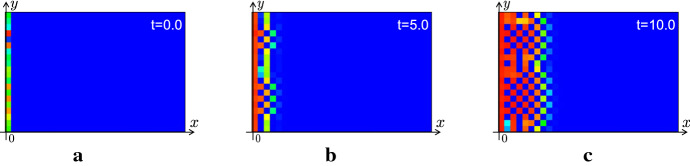
The results of the numerical simulations for the state of the differentiation $A_{i,j}$ in the discrete model (31) on the square lattice $\{ c_{i,j}\}$. The parameters are $d_e=0.5, k_e=1.0, a_e=1.0, k_n=2.0, d_t=2.0, d_c=0.1, k_d=1.5, a_d=1.0, A_0=1.0$, and $e_a=10.0$

## Appendix A

### Numerical results of the discrete model of PW

In this appendix we will show the numerical results of the original discrete model of PW (31). The results is shown in Fig. 17.

The propagation of the salt-and-pepper pattern, and stripe pattern are observed in the numerical simulations. We see that the profile of $A_{i,j}$ are uniform in each cell $c_{i,j}$.

## Appendix B

### Energy estimate

We estimate error between the solutions of ($\hbox {P}_S$) and ($\hbox {P}_\varepsilon$) with (11) in the singular limit analysis with linear function *f* given by (10). We assume the initial condition of $(P_S)$ and $(P_{\varepsilon })$ is the same, and given by $u(x,0)=u^\varepsilon (x,0) = u_0(x) = \sum _{i=1 }^{N } u_{i}(0) \chi _{c_i}(x) \in L^\infty ({\mathbb {T}})$.

First, we compute the fundamental solution of ($\hbox {P}_S$) without *g*(*u*). As the equation ($\hbox {P}_S$) and the equation ($\hbox {P}_D$) is equivalent, we solve the equation ($\hbox {P}_D$). The system ($\hbox {P}_D$) is described by

42 $$\begin{aligned} V_t = A V + F(V), \end{aligned}$$

where $$V=(u_1(t), \ldots , u_N(t))$$, $$F: {\mathbb {R}}^N \rightarrow {\mathbb {R}}^N$$ and

$$\begin{aligned} A&= \begin{pmatrix} a_0 &{}\quad a_1 &{}\quad \cdots &{}\quad a_{ [\frac{N-1}{2}] } &{}\quad a_{ -[ \frac{N-1}{2}] } &{}\quad \cdots &{}\quad a_{-1} \\ a_{-1} &{}\quad a_0 &{}\quad a_1 &{}\quad \cdots &{}\quad &{}\quad &{}\quad \vdots \\ \vdots &{}\quad \ddots &{}\quad \ddots &{}\quad \ddots &{}\quad &{}\quad &{}\quad \vdots \\ \vdots &{}\quad &{}\quad \ddots &{}\quad \ddots &{}\quad \ddots &{}\quad &{}\quad \vdots \\ \vdots &{}\quad &{}\quad &{}\quad \ddots &{}\quad \ddots &{}\quad \ddots &{}\quad \vdots \\ \vdots &{}\quad &{}\quad &{}\quad \cdots &{}\quad a_{-1} &{}\quad a_0 &{}\quad a_1 \\ a_1 &{}\quad \cdots &{}\quad a_{ [\frac{N-1}{2}] } &{}\quad a_{ -[ \frac{N-1}{2}] } &{}\quad \cdots &{}\quad a_{-1} &{} \quad a_0 \end{pmatrix},\ (if\ N\ is\ odd), \\ \end{aligned}$$

$$\begin{aligned} A&= \begin{pmatrix} a_0 &{}\quad a_1 &{}\quad \cdots &{}\quad a_{ [\frac{N-1}{2}] } &{}\quad 0 &{}\quad a_{ -[ \frac{N-1}{2}] } &{}\quad \cdots &{}\quad a_{-1} \\ a_{-1} &{}\quad a_0 &{}\quad a_1 &{}\quad \cdots &{}\quad &{}\quad &{}\quad &{}\quad \vdots \\ \vdots &{}\quad \ddots &{}\quad \ddots &{}\quad \ddots &{}\quad &{}\quad &{}\quad &{}\quad \vdots \\ \vdots &{}\quad &{}\quad \ddots &{}\quad \ddots &{}\quad \ddots &{}\quad &{}\quad &{}\quad \vdots \\ \vdots &{}\quad &{}\quad &{}\quad \ddots &{}\quad \ddots &{}\quad \ddots &{}\quad &{}\quad \vdots \\ \vdots &{}\quad &{}\quad &{}\quad &{}\ddots &{}\quad \ddots &{}\quad \ddots &{}\quad \vdots \\ \vdots &{}\quad &{}\quad &{}\quad &{}\quad \cdots &{}\quad a_{-1} &{}\quad a_0 &{}\quad a_1 \\ a_1 &{}\quad \cdots &{}\quad a_{ [\frac{N-1}{2}] } &{}\quad 0 &{}\quad a_{ -[ \frac{N-1}{2}] } &{}\quad \cdots &{}\quad a_{-1} &{}\quad a_0 \end{pmatrix},\ (if\ N\ is\ even). \end{aligned}$$

Since the matrix *A* is cyclic, we obtain the eigenvalue (12) i.e.,

$$\begin{aligned} \lambda _k = \sum _{j=-[\frac{N-1}{2}] }^{[\frac{N-1}{2}]} a_j \omega ^{ jk}. \end{aligned}$$

Associated eigenvector $V_k$ of which components are $\{ v_n \}_{n=1}^N$ with eigenvalue $\lambda _k$ is computed by $v_n = \omega ^{nk}, \ (k=1,\ldots , N)$. Thus, the fundamental solution of (42) is given by

$$\begin{aligned} V(t) = \sum _{k=1}^N p_k e^{\lambda _k t} V_k = \sum _{k=1}^N p_k e^{\lambda _k t} \begin{pmatrix} \omega ^k \\ \omega ^{2k} \\ \vdots \\ \omega ^{Nk} \end{pmatrix}, \end{aligned}$$

where $\{p_k\}^N_{k=1}$ are constants satisfying

$$\begin{aligned} \begin{pmatrix} p_1 \\ p_2 \\ \vdots \\ \vdots \\ p_N \end{pmatrix} = { \frac{1}{N} \begin{pmatrix} \omega &{}\omega ^{-2} &{}\cdots &{}\omega ^{-N} \\ \omega ^{-2} &{}\omega ^{-4} &{}\cdots &{}\omega ^{-2N} \\ \vdots &{}\vdots &{}\ddots &{} \vdots \\ \vdots &{}\vdots &{} &{}\vdots \\ \omega ^{-N} &{}\omega ^{-2N} &{}\cdots &{}\omega ^{-N^2} \end{pmatrix} } \begin{pmatrix} u_1(0) \\ u_2(0) \\ \vdots \\ \vdots \\ u_N(0) \end{pmatrix}. \end{aligned}$$

Therefore, we obtain the exact solution as

$$\begin{aligned} u(x, t ) = \sum _{j=1}^N u_j(t) \chi _{c_j}(x) = \sum _{j=1}^N \sum _{k=1}^N p_k e^{ \lambda _k t} \omega ^{jk} \chi _{c_j}(x). \end{aligned}$$

Setting a vector ${\tilde{V}}_k$ as $\{ {\tilde{v}}_n \}= \omega ^{-nk}$ and ${\tilde{\lambda }}_k = \sum _{j=-[(N-1)/2] }^{[ (N-1)/2] } a_j \omega ^{ -j k }$, we find that $A{\tilde{V}}_k = \tilde{\lambda _k}{\tilde{V}}_k$. Thus, if $a_j = a_{-j}, \ ( j= 1, \ldots , [\frac{N-1}{2}])$, we obtain that $\lambda _k = a_0 + 2 \sum _{j= 1 }^{[(N-1)/2 ]} a_j \cos \Big ( \frac{ 2\pi {j} k}{N} \Big )$, $A( V_k + {\tilde{V}}_k ) = \lambda _k V_k + {\tilde{\lambda }}_k {\tilde{V}}_k= \lambda _k ( V_k + {\tilde{V}}_k)$, and $A( V_k - {\tilde{V}}_k ) = \lambda _k V_k - {\tilde{\lambda }}_k {\tilde{V}}_k= \lambda _k ( V_k - {\tilde{V}}_k)$. From this calculation, setting a vector $W_k$ as $\{w_n \}=\cos (2\pi nk/N)+\sin (2\pi nk/N)$. Then $AW_k=\lambda _k W_k$. Thus the fundamenal solution of (42) is given by

$$\begin{aligned} V(t)=\sum ^{ N }_{k=1} q_k e^{\lambda _k t} W_k, \end{aligned}$$

where $\{q_k\}_{k=1}^{ N }$ are constants satisfying

$$\begin{aligned} \begin{pmatrix} q_1 \\ q_2 \\ \vdots \\ \vdots \\ q_N \end{pmatrix} ={\frac{1}{N} (W_1, W_2, \ldots , W_N)^t } \begin{pmatrix} u_1(0) \\ u_2(0) \\ \vdots \\ \vdots \\ u_N(0) \end{pmatrix}, \end{aligned}$$

where the matrix $(W_1, W_2, \ldots , W_N)^t$ is the transpose of the matrix $(W_1, W_2, \ldots , W_N)$.

Next, we show the existence the global solution of ($\hbox {P}_S$) and ($\hbox {P}_\varepsilon$) under the above assumptions, respectively.

#### Proof of Proposition 2

The existence and uniqueness of the mild solution for ($\hbox {P}_S$) in $C([0,T], L^\infty ({\mathbb {T}}))$ is guaranteed by the fixed point theorem. We show the global existence in $L^\infty ({\mathbb {T}})$ by the argument of the maximum principle. For a contradiction, we assume that there exists a positive finite constant $T>0$ such that

$$\begin{aligned} \limsup _{\tau \nearrow T} \max _{0\le t \le \tau } \left\| u(\cdot ,t) \right\| _{L^\infty ({\mathbb {T}})} = \infty \end{aligned}$$

Then we take a sequence $\{ T_n \}_{n \in N}, \ T_n \nearrow T$ as $n \rightarrow \infty$ such that

$$\begin{aligned} R_n : = \max _{0\le t \le T_n} \left\| u(\cdot ,t) \right\| _{L^\infty ({\mathbb {T}})} \rightarrow \infty \end{aligned}$$

as $n \rightarrow \infty$. Indeed, for any *R* there exists a positive constant $n_0 \in {\mathbb {N}}$ such that for all $n \ge n_0$, we see that $R_n > R$, and for any $0<t<T_n$, there exist $j_n \in {\mathbb {N}}$ such that $\left\| u(\cdot , t) \right\| _{L^\infty ({\mathbb {T}})} = |u_{j_n}(t)|$. We define the points $(x_n, t_n) \in {\mathbb {T}}\times (0, T_n)$ as satisfying $|u(x_n, t_n)| = | u_{j_n}(t_n) | = R_n$. As $R_n \rightarrow \infty$, we can choose

$$\begin{aligned} \frac{\partial }{\partial t}(u^2)(x_n, t_n) \ge 0. \end{aligned}$$

Multiplying the principal equation of ($\hbox {P}_S$) by *u*, then

$$\begin{aligned} 0 \le \frac{1}{2}\frac{\partial }{\partial t}(u^2)(x_n, t_n)&=\sum _{j=-\left[ \frac{N-1}{2}\right] }^{\left[ \frac{N-1}{2}\right] } a_ju(x_n + jl,t_n)u(x_n,t_n)+g( u(x_n, t_n))u(x_n,t_n), \\&\le \left( \sum _{j=-\left[ \frac{N-1}{2}\right] }^{\left[ \frac{N-1}{2}\right] } |a_j| + g_2 \right) | u(x_n, t_n) |^{2} -g_0 | u(x_n, t_n) |^{p+1} + g_1 | u(x_n, t_n) |^3 , \\&= \left( \sum _{ j = -\left[ \frac{N-1}{2}\right] }^{\left[ \frac{N-1}{2}\right] } |a_j| + g_2 \right) R^2_n -g_0 R^{p+1}_n + g_1 R^3_n \rightarrow -\infty ,\quad (n\rightarrow \infty ), \end{aligned}$$

from (A2) and considering the degree of polynomial of $R_n$. This yields a contradiction. $\square$

Similar to the proof of Proposition 2, we show the global existence of the equation ($\hbox {P}_\varepsilon$).

#### Proof of Proposition 3

The existence and uniqueness of the mild solution for ($\hbox {P}_\varepsilon$) in $C([0,T], L^\infty ({\mathbb {T}}))$ is guaranteed by the fixed point theorem. By the same argument as the proof of Proposition 2, we obtain the $L^\infty ({\mathbb {T}})$ estimate for the solution of ($\hbox {P}_\varepsilon$). For a contradiction, we assume that there exists a positive finite constant $T>0$ such that

$$\begin{aligned} \limsup _{\tau \nearrow T} \max _{0\le t \le \tau } \left\| u^\varepsilon (\cdot ,t) \right\| _{L^\infty ({\mathbb {T}})} = \infty \end{aligned}$$

Then we take a sequence $\{ T_n \}_{n \in N}, \ T_n \nearrow T$ as $n \rightarrow \infty$ such that

$$\begin{aligned} R_n : = \max _{0\le t \le T_n} \left\| u^\varepsilon (\cdot ,t) \right\| _{L^\infty ({\mathbb {T}})} \rightarrow \infty \end{aligned}$$

as $$n \rightarrow \infty $$. Indeed, for any *R* there exists a positive constant $$n_0 \in {\mathbb {N}}$$ such that for all $$n \ge n_0$$, we have $$R_n > R$$, and for any $$0<t<T_n$$, we define the points satisfying $$|u^\varepsilon (x_n, t_n)| = R_n$$ by $$(x_n, t_n) \in {\mathbb {T}}\times (0, T_n)$$. In the case that the candidates of the maximum point is on the discontinuous point, employing the larger value at the point by taking the left-sided and right-sided limits, we define the point as $$(x_n, t_n) $$. As $$R_n \rightarrow \infty $$, we can choose

$$\begin{aligned} \frac{\partial }{\partial t}[(u^\varepsilon )^2](x_n, t_n) \ge 0. \end{aligned}$$

Moreover, for any $\{ a_j \}_{j=1}^N$, and positive constants $p, g_0, g_1$ and $g_2$, there exist sufficient large constant *r*, we see that

$$\begin{aligned} \left\| K \right\| _{L^1({\mathbb {T}})} r^2 +g(r)r \le \left( \sum _{ j = - \left[ \frac{N-1}{2}\right] } ^ { \left[ \frac{N-1}{2}\right] } |a_j| +g_2 \right) |r|^2 -g_0 |r|^{p+1} + g_1|r|^3 < 0 \end{aligned}$$

from (A2) and considering the degree of polynomial of *r*. Thus, there exists a positive constant $n_1 \in N$ such that for $n\ge n_1$

$$\begin{aligned}&0 \le \frac{1}{2}\frac{\partial }{\partial t}[(u^\varepsilon )^2](x_n, t_n) \\&\quad \le \left( \sum _{ j = - \left[ \frac{N-1}{2}\right] }^ { \left[ \frac{N-1}{2}\right] } |a_j| + g_2 \right) | u^\varepsilon (x_n, t_n) |^2 -g_0 | u^\varepsilon (x_n, t_n) |^{p+1} + g_1 | u^\varepsilon (x_n, t_n) |^3 < 0, \end{aligned}$$

replacing *r* with $u^\varepsilon (x_n, t_n)$. This yields a contradiction. $\square$

Now we show the singular limit analysis between the solutions of ($\hbox {P}_S$) and ($\hbox {P}_\varepsilon$).

#### Proof (Proof of Theorem 1)

Taking the difference between the solutions of ($\hbox {P}_S$) and ($\hbox {P}_\varepsilon$), we have

$$\begin{aligned} U^\varepsilon _t =\sum _{j=-[\frac{ N-1 }{2}] }^{ [\frac{ N-1 }{2}] } a_j \int _{{\mathbb {T}}} u(x + jl,t) - (\rho _{\varepsilon ,jl}*u^\varepsilon ) dx + g(u)-g(u^\varepsilon ). \end{aligned}$$

Multiplying the above equation by $U^\varepsilon$ and integrating it with respect to *x*, we obtain that

$$\begin{aligned} \begin{aligned} \frac{1}{2} \frac{d}{dt} \left\| U^\varepsilon \right\| _{L^2({\mathbb {T}})} ^2&= \sum _{j=-[\frac{ N-1 }{2}]}^{ [\frac{ N-1 }{2}] } a_j \int _{{\mathbb {T}}} \left\{ u(x + jl,t) - (\rho _{\varepsilon ,jl}*u^\varepsilon ) \right\} U^\varepsilon dx + \int _{{\mathbb {T}}} (g(u)-g(u^\varepsilon ))U^\varepsilon dx \\&\le \sum _{j=-[ \frac{ N-1 }{2}]}^{ [ \frac{ N-1 }{2}] } |a_j| \int _{{\mathbb {T}}} \left\{ u(x + jl,t) - (\rho _{\varepsilon ,jl}*u^\varepsilon ) \right\} U^\varepsilon dx \\&\quad + \int _{{\mathbb {T}}} (-g_0p|u+\theta u^\varepsilon |^{p-1} +g_3|u+\theta u^\varepsilon | +g_4 )(U^\varepsilon )^2 dx \\&\le \sum _{j=-[\frac{ N-1 }{2}]}^{ [\frac{ N-1 }{2}] } |a_j| \int _{{\mathbb {T}}} \left\{ u(x + jl,t) - (\rho _{\varepsilon ,jl}*u^\varepsilon ) \right\} U^\varepsilon dx +C_{7}\left\| U^\varepsilon \right\| _{L^2({\mathbb {T}})} ^2 \end{aligned} \end{aligned}$$

from (A3) and where $\theta \in (0,1)$, and $C_{7}=( g_3 \sup _{t >0} \Vert u+\theta u^\varepsilon \Vert _{L^\infty ({\mathbb {T}})} +g_4 )$. Here the intercellular interaction can be computed as follows:

$$\begin{aligned}&\int _{\mathbb {T}}( (u^\varepsilon *\rho _{\varepsilon , jl})-u( y + jl, t) )U^\varepsilon dy \\&\quad =\int _{\mathbb {T}}( ( u^\varepsilon *\rho _{\varepsilon } ) (y + jl, t)-u( y + jl, t) )U^\varepsilon dy\\&\quad \le \frac{1}{2} \int _{\mathbb {T}}(( u^\varepsilon *\rho _{\varepsilon } )(y + jl, t )-u( y + jl, t) )^2 dy + \frac{1}{2} \left\| U^\varepsilon \right\| _{L^2({\mathbb {T}})}^2\\&\quad = \frac{1}{2} \int _{\mathbb {T}}( (u^\varepsilon *\rho _{\varepsilon } )(y,t)-u(y,t) )^2 dy + \frac{1}{2} \left\| U^\varepsilon \right\| _{L^2({\mathbb {T}})}^2\\&\quad \le \int _{\mathbb {T}}( U^\varepsilon * \rho _\varepsilon )^2 dy + \int _{\mathbb {T}}(u*\rho _\varepsilon - u)^2 dy + \frac{1}{2} \left\| U^\varepsilon \right\| _{L^2({\mathbb {T}})}^2. \end{aligned}$$

for $j=-[\frac{ N-1 }{2}], \ldots , -1, 0, 1, \ldots , [\frac{ N-1 }{2}]$. Using the Hölder inequality and the property of the mollifier, we estimate that

$$\begin{aligned} \int _{\mathbb {T}}(u*\rho _\varepsilon - u)^2 dy&= \int _{\mathbb {T}}\Big \{ \int _{\mathbb {T}}\Big ( u( y-z, t ) - u(y, t) \Big ) \rho _\varepsilon (z) dz \Big \}^2 dy \\&\le \int _{\mathbb {T}}\rho _\varepsilon (z) dz \int _{\mathbb {T}}\int _{\mathbb {T}}\Big ( \tau _z u - u \Big )^2 \rho _\varepsilon (z) dz dy \\&= \int _{B(0, \varepsilon )} \left\| \tau _z u - u \right\| _{L^2({\mathbb {T}})}^2 \rho _\varepsilon (z) dz \\&\le \sup _{ |z|<\varepsilon , t >0 }\left\| \tau _z u - u \right\| _{L^2({\mathbb {T}})}^2. \end{aligned}$$

Therefore, we obtain that

$$\begin{aligned} \int _{\mathbb {T}}( U^\varepsilon * \rho _\varepsilon )^2 dy + \int _{\mathbb {T}}(u*\rho _\varepsilon - u)^2 dy \le \left\| U^\varepsilon \right\| _{L^2({\mathbb {T}})} ^2 + \sup _{ |z|<\varepsilon , t >0 }\left\| \tau _z u - u \right\| _{L^2({\mathbb {T}})}^2. \end{aligned}$$

Combining the above calculations, we have

$$\begin{aligned} \frac{1}{2} \frac{d}{dt} \left\| U^\varepsilon \right\| _{L^2({\mathbb {T}})} ^2 \le \sum _{j=-[\frac{ N-1 }{2}]}^{ [\frac{ N-1 }{2}] } |a_j| \left( \frac{3}{2} \left\| U^\varepsilon \right\| _{L^2({\mathbb {T}})} ^2 + \sup _{ |z|<\varepsilon , t >0 }\left\| \tau _z u - u \right\| _{L^2({\mathbb {T}})}^2 \right) + C_{7} \left\| U^\varepsilon \right\| _{L^2({\mathbb {T}})} ^2. \end{aligned}$$

Utilizing the classical Gronwall lemma yields that

$$\begin{aligned} \left\| U^\varepsilon \right\| _{L^2({\mathbb {T}})} ^2 \le \frac{ C_{8} }{C_{7}} \sup _{|y| < \varepsilon , t>0} \Vert \tau _y u - u \Vert _{L^2({\mathbb {T}})}^2 \left( e^{C_{7} T} -1 \right) , \end{aligned}$$

where

$$\begin{aligned} C_{8} = 3 \sum _{j=-[\frac{ N-1 }{2}]}^{ [\frac{ N-1 }{2}] } |a_j| + 2C_{7}, \quad C_{9} = 2 \sum _{j=-[\frac{ N-1 }{2}]}^{ [\frac{ N-1 }{2}] } |a_j|. \end{aligned}$$

$$\square $$

## Appendix C

### Derivation on square and hexagonal lattices

In this appendix we put the detailed calculations for the derivations of the continuous models in square and hexagonal lattices. For (25), we can calculate as

$$\begin{aligned} \sum _{i,j=1}^N u_{i-p, j-q} \chi _{c_{i,j}}(x,y ) = u(x- pl_x, y-ql_y,t), \quad (p,q \in \{1, \ldots , N \}). \end{aligned}$$

Thus, we have

$$\begin{aligned} u_{t}&= f \left( \sum _{i,j=1}^N u_{i-1, j} \chi _{c_{i,j}}(x,y ), \ \sum _{i,j=1}^N u_{i+1, j} \chi _{c_{i,j}}(x,y ), \ u, \right. \\&\qquad \quad \left. \sum _{i,j=1}^N u_{i, j-1}\chi _{c_{i,j}}(x,y ), \ \sum _{i,j=1}^N u_{i, j+1} \chi _{c_{i,j}}(x,y ) \right) +g(u)\\&= f ( u(x-l_x,y,t), \ u(x+l_x,y,t), \ u(x,y,t), \ u(x, y-l_y, t), \ u(x,y+l_y, t) )+g( u ). \end{aligned}$$

The discrete model is successfully converted into the continuous model. Similarly to the case in one dimension, approximating the shift operator by the convolution with the mollifier yields the nonlocal evolution equation:

$$\begin{aligned} u_{t}^\varepsilon&= f ( (\tau _{l_x,0}{\varvec{\rho }}_\varepsilon ) * u^\varepsilon , \ (\tau _{-l_x,0}{\varvec{\rho }}_\varepsilon ) * u^\varepsilon , \ u^\varepsilon , \ (\tau _{0,l_y}{\varvec{\rho }}_\varepsilon ) * u^\varepsilon , \ (\tau _{0, -l_y}{\varvec{\rho }}_\varepsilon ) * u^\varepsilon )+g( u^\varepsilon ), \end{aligned}$$

where we define the shift operator $\tau _{l,m}$ as

$$\begin{aligned} \tau _{l,m} u = u(x-l, y-m). \end{aligned}$$

If *f* is linear, the description with the kernel is given as follows

$$\begin{aligned} u_{t}^\varepsilon&= f ( (\tau _{l_x,0}{\varvec{\rho }}_\varepsilon ) * u^\varepsilon , \ (\tau _{-l_x,0}{\varvec{\rho }}_\varepsilon ) * u^\varepsilon , \ u^\varepsilon , \ (\tau _{0,l_y}{\varvec{\rho }}_\varepsilon ) * u^\varepsilon , \ (\tau _{0, -l_y}{\varvec{\rho }}_\varepsilon ) * u^\varepsilon )+g( u^\varepsilon )\\&= K * u^\varepsilon + a_0 u^\varepsilon +g( u^\varepsilon ), \end{aligned}$$

where

$$\begin{aligned} K= a_{-2} (\tau _{l_x,0}{\varvec{\rho }}_\varepsilon )+ a_{-1} (\tau _{-l_x,0}{\varvec{\rho }}_\varepsilon ) + a_1 (\tau _{0,l_y}{\varvec{\rho }}_\varepsilon ) + a_2 (\tau _{0,-l_y}{\varvec{\rho }}_\varepsilon ). \end{aligned}$$

In the case of hexagonal lattice, the derivation is given as follows. Here for simple description, introducing the complex variable ${\varvec{x}}=x+y i \in {\mathbb {C}}$, we identify the two-dimensional Euclid space ${\mathbb {R}}^2$ with the complex plane ${\mathbb {C}}$. Then we compute that

$$\begin{aligned} \begin{aligned} \sum _{ j=1 }^N u_{\varLambda _j^k }(t) \chi _{c_ j} (x,y)&= u\Big ( {\varvec{x}} + l e^{i(\frac{\pi }{2} - \frac{ (k-1) \pi }{3})}, t \Big )\\&= u\Big (x + \cos \Big (\frac{\pi }{2} - \frac{\pi (k-1)}{3} \Big ) l, y+ \sin \Big (\frac{\pi }{2} - \frac{\pi (k-1)}{3}\Big ) l, t \Big ). \end{aligned} \end{aligned}$$

Changing the variable through the characteristic function (27), we obtain the continuous model:

$$\begin{aligned} \begin{aligned} u_t = f\Big (&u( {\varvec{x}} + l e^{i \frac{\pi }{2} }, t ), u( {\varvec{x}} + l e^{i(\frac{\pi }{2} - \frac{ \pi }{3})}, t ), u( {\varvec{x}} + l e^{i(\frac{\pi }{2} - \frac{2 \pi }{3})}, t ), \\&u( {\varvec{x}} + l e^{i(\frac{\pi }{2} - \frac{3 \pi }{3})}, t ), u( {\varvec{x}} + l e^{i(\frac{\pi }{2} - \frac{4 \pi }{3})}, t ), u( {\varvec{x}} + l e^{i(\frac{\pi }{2} - \frac{5 \pi }{3})}, t ), u\Big ) +g(u)\\ = f\Big (&u(x, y+l,t), u \Big (x+\frac{\sqrt{3}}{2}l, y + \frac{1}{2}l, t \Big ), u \Big ( x+\frac{\sqrt{3}}{2}l, y - \frac{1}{2}l, t \Big ), \\&u(x, y-l,t), u\Big ( x-\frac{\sqrt{3}}{2}l, y - \frac{1}{2}l, t \Big ), u \Big (x-\frac{\sqrt{3}}{2}l, y + \frac{1}{2}l, t\Big ), u(x,y,t)\Big ) +g(u). \end{aligned} \end{aligned}$$

Using the shift operator (28) and approximating the shift operator by the convolution with the mollifier, we can derive the kernel corresponding to the intercellular interaction on the hexagonal lattice.

$$\begin{aligned} u_t^\varepsilon&= f\Big ( (\tau _{\varLambda ^1} {\varvec{\rho }}_\varepsilon )*u^\varepsilon , (\tau _{\varLambda ^2} {\varvec{\rho }}_\varepsilon )*u^\varepsilon , (\tau _{\varLambda ^3} {\varvec{\rho }}_\varepsilon )*u^\varepsilon , \\&\qquad \quad (\tau _{\varLambda ^4} {\varvec{\rho }}_\varepsilon )*u^\varepsilon , (\tau _{\varLambda ^5} {\varvec{\rho }}_\varepsilon )*u^\varepsilon , (\tau _{\varLambda ^6} {\varvec{\rho }}_\varepsilon )*u^\varepsilon , u^\varepsilon \Big )+g(u^\varepsilon )\\&= \Big (\sum _{k=1}^6 a_k (\tau _{\varLambda ^k} {\varvec{\rho }}_\varepsilon ) \Big ) * u^\varepsilon + a_0 u^\varepsilon +g(u^\varepsilon )\\&=K*u^\varepsilon +a_0 u^\varepsilon + g(u^\varepsilon ), \end{aligned}$$

where

$$\begin{aligned} K=K(x,y)=\sum _{k=1}^6 a_k (\tau _{\varLambda ^k} {\varvec{\rho }}_\varepsilon ). \end{aligned}$$

## Appendix D

### Reaction, diffusion and nonlocal interaction on growing domain

We explain the notion of the mathematical model on a growing domain by using a reaction diffusion equation with nonlocal interactions. Based on the previous reports (Crampin et al. 1999, 2002), general scalar reaction diffusion equation with convolution term on a growing domain in one-dimensional space is given by

43 $$\begin{aligned} \dfrac{\partial u}{\partial t}+\nabla (a\cdot u)=\varDelta u+K*u+f(u), \quad {\mathrm {in}}\ (0, L(t))\times \{t>0\}, \end{aligned}$$

where $u=u(t,x)$ is an unknown function, $f: {\mathbb {R}}\rightarrow {\mathbb {R}}$ is a nonlinear function, $a=a(x,t)$ is the velocity field of the flow and satisfies

$$\begin{aligned} \frac{dx}{dt}=a(x,t),\quad x\in (0,L(t)). \end{aligned}$$

Now, we assume that there exists the continuous derivative bijection function $\varGamma : (0, L(0)) \rightarrow (0, L(t))$ for all $t>0$. Then, $x\in (0,L(t))$ is described by

44 $$\begin{aligned} x=\varGamma (y,t),\quad y\in (0,L(0)). \end{aligned}$$

Using (44), we take the change of variables from $x\in (0,L(t))$ to $y\in (0,L(0))$

45 $$\begin{aligned} \dfrac{\partial u}{\partial t}+ \eta u=\frac{1}{\varGamma _y}\frac{\partial }{\partial y}\left( \frac{1}{\varGamma _y}\frac{\partial u}{\partial y}\right) +{\tilde{K}}{*}u+f(u), \quad {\mathrm {in}}\ (0, L(0)), \end{aligned}$$

where $\eta :=a_x$, and ${\tilde{K}}$ is the integral operator satisfing

$$\begin{aligned} {\tilde{K}}{*}u(y,t):=\int ^{L(0)}_{0} \varGamma _y(s,t)K(\varGamma (y,t)-\varGamma (s,t))u(s,t) ds. \end{aligned}$$

To calculate $\varGamma _y$, we deduce the time evolutional equation of $\varGamma _y$. Setting the velocity field as

$$\begin{aligned} a=\left. \frac{\partial \varGamma }{\partial t}\right| _{y} (y,t), \end{aligned}$$

then we can compute that

46 $$\begin{aligned} \frac{\partial \varGamma _y}{\partial t}=\eta (y,t)\varGamma _y \end{aligned}$$

by using the chain rule. Moreover, we impose the following initial and boundary condition

47 $$\begin{aligned} \varGamma (y,0)=y,\ (y\in (0,L(0))),\quad \varGamma (0,t)=0, \ (t>0). \end{aligned}$$

From (46) and (47), we can calculate (45) numerically, if we define the function $\eta$.

## Appendix E

### Spectrum method on sphere surface

We explain the basic idea of spectrum method for the numerical calculation on the unit sphere. Set ${\mathbb {S}}^2:=\{x=(x_1,x_2,x_3)\in {\mathbb {R}}^3\mid \ |x|=1\}$, and let $x\in {\mathbb {S}}^2$ be a point on the sphere parameterized by the $(\lambda , \theta )\in (-1, 1)\times (0, 2\pi )$ as

$$\begin{aligned} x= \left( \begin{array}{c} x_1\\ x_2\\ x_3 \end{array} \right) = \left( \begin{array}{c} \sqrt{1-\lambda ^2}\cos \theta \\ \sqrt{1-\lambda ^2}\sin \theta \\ \lambda \end{array} \right) . \end{aligned}$$

Moreover, denoting the Euclidian distance in ${\mathbb {R}}^3$ and the standard measure on ${\mathbb {S}}^2$ by $|\cdot |$ and $d\mu$, respectively. To explain the spectrum method, we consider the following equation:

48 $$\begin{aligned} \dfrac{\partial u}{\partial t}=\varDelta _{{\mathbb {S}}^2}u+K*_{{\mathbb {S}}^2}u+f(u), \quad {\mathrm {in}}\ {\mathbb {S}}^2 \times \{ t>0\}, \end{aligned}$$

where $u=u(x,t)$ is an unknown function, $f: {\mathbb {R}}\rightarrow {\mathbb {R}}$ is a nonlinear function, the Laplace-Beltrami operator on the unit sphere $\varDelta _{{\mathbb {S}}^2}$ is described by

$$\begin{aligned} \varDelta _{{\mathbb {S}}^2}u=\dfrac{1}{1-\lambda ^2}\dfrac{\partial ^2 u}{\partial \theta ^2}+\dfrac{\partial }{\partial \lambda }\left\{ (1-\lambda ^2)\dfrac{\partial u}{\partial \lambda } \right\} , \end{aligned}$$

and the $*_{{\mathbb {S}}^2}$ denotes the convolution operator with respective to the spatial variable as

$$\begin{aligned} K*_{{\mathbb {S}}^2}u(x,t):=\int _{{\mathbb {S}}^2}K(|x-y|)u(y,t)d\mu (y), \end{aligned}$$

and we suppose that the kernel $K: [0, 2]\rightarrow {\mathbb {R}}$ satisfies $K(\sqrt{2(1-\cdot )})\in L^1(-1,1)$.

To calculate the diffusion term and the convolution term on the sphere surface, we introduce the spherical harmonics. The spherical harmonics $Y^m_n=Y^m_n(\lambda , \theta )(n=0, 1, 2\cdots , |m|\le n)$ are given by

$$\begin{aligned} Y^m_n(\lambda , \theta )&:=\frac{C^{|m|}_n}{\sqrt{2\pi }}e^{im\theta }P^{|m|}_n(\lambda ), \\ P^m_n(\lambda )&:=\dfrac{1}{2^n n!}(1-\lambda ^2)^{m/2}\dfrac{d^{m+n}}{d\lambda ^{m+n}}\left[ (\lambda ^2-1)^n\right] , \quad (m\ge 0), \\ C^m_n&:=\sqrt{\left( n+\frac{1}{2}\right) \frac{(n-m)!}{(n+m)!}}, \quad (m\ge 0), \end{aligned}$$

where $P^m_n(\lambda )$ is the associated Legendre polynomials, and $C^m_n$ satisfies $C^m_n=\left( || P^m_n ||_{L^2(-1,1)}\right) ^{-1/2}$. The spherical harmonics have the following properties :

#### Proposition 4

(Atkinson and Han 2012)

1. $\{Y^m_n\}$ is complete orthonormal system on $L^2({\mathbb {S}}^2)$.
2. $$\varDelta _{{\mathbb {S}}^2}Y^m_n=-n(n+1)Y^m_n.$$
3. $$K*_{{\mathbb {S}}^2}Y^m_n=2\pi \beta _n Y^m_n$$, where $$\beta _n=\int ^1_{-1}K(\sqrt{2(1-s)})P^0_n(s)ds$$.

By this proposition, we can expand *u* and *f*(*u*) by spherical harmonics

$$\begin{aligned} u(x,t)&=\sum ^{\infty }_{n=0} \sum ^{n}_{m=-n}u^m_n(t)Y^m_n(x)\simeq \sum ^{N}_{n=0} \sum ^{n}_{m=-n}u^m_n(t)Y^m_n(x),\\ f(u)(x,t)&=\sum ^{\infty }_{n=0} \sum ^{n}_{m=-n}f^m_n(t)Y^m_n(x)\simeq \sum ^{N}_{n=0} \sum ^{n}_{m=-n}f^m_n(t)Y^m_n(x), \end{aligned}$$

where *N* is a sufficiently large natural number, $u^m_n(t)=\left<u(t), Y^m_n \right>_{L^2({\mathbb {S}}^2)}$, $f^m_n(t)=\left<f(u)(t), Y^m_n \right>_{L^2({\mathbb {S}}^2)}$. Therefore, calculating (48) yields that

$$\begin{aligned} \frac{du^m_n}{dt}=\{-n(n+1)+\beta _n \}u^m_n+f^m_n, \quad (n=0, 1, 2\cdots , N, |m|\le n) \end{aligned}$$

for the every time step, since $f^m_n$ depends on *u*.
